# Effect of Phosphorylated Tau on Cortical Pyramidal Neuron Morphology during Hibernation

**DOI:** 10.1093/texcom/tgaa018

**Published:** 2020-05-21

**Authors:** Mamen Regalado-Reyes, Ruth Benavides-Piccione, Isabel Fernaud-Espinosa, Javier DeFelipe, Gonzalo León-Espinosa

**Affiliations:** Laboratorio Cajal de Circuitos Corticales, Centro de Tecnología Biomédica, Universidad Politécnica de Madrid, Madrid 28002, Spain; Laboratorio Cajal de Circuitos Corticales, Centro de Tecnología Biomédica, Universidad Politécnica de Madrid, Madrid 28002, Spain; Instituto Cajal, Consejo Superior de Investigaciones Científicas, Madrid 28002, Spain; Laboratorio Cajal de Circuitos Corticales, Centro de Tecnología Biomédica, Universidad Politécnica de Madrid, Madrid 28002, Spain; Laboratorio Cajal de Circuitos Corticales, Centro de Tecnología Biomédica, Universidad Politécnica de Madrid, Madrid 28002, Spain; Instituto Cajal, Consejo Superior de Investigaciones Científicas, Madrid 28002, Spain; Laboratorio Cajal de Circuitos Corticales, Centro de Tecnología Biomédica, Universidad Politécnica de Madrid, Madrid 28002, Spain; Facultad de Farmacia, Departamento de Química y Bioquímica, Universidad San Pablo-CEU, CEU Universities, Urbanización Montepríncipe, Boadilla del Monte, Madrid 28660, Spain

**Keywords:** 3D reconstructions, cerebral cortex, dendrites, dendritic spines, Syrian hamster

## Abstract

The dendritic spines of pyramidal cells are the main postsynaptic target of excitatory glutamatergic synapses. Morphological alterations have been described in hippocampal dendritic spines during hibernation—a state of inactivity and metabolic depression that occurs via a transient neuronal tau hyperphosphorylation. Here, we have used the hibernating Syrian hamster to investigate the effect of hyperphosphorylated tau regarding neocortical neuronal structure. In particular, we examined layer Va pyramidal neurons. Our results indicate that hibernation does not promote significant changes in dendritic spine density. However, tau hyperphosphorylated neurons show a decrease in complexity, an increase in the tortuosity of the apical dendrites, and an increase in the diameter of the basal dendrites. Tau protein hyperphosphorylation and aggregation have been associated with loss or alterations of dendritic spines in neurodegenerative diseases, such as Alzheimer’s disease (AD). Our results may shed light on the correlation between tau hyperphosphorylation and the neuropathological processes in AD. Moreover, we observed changes in the length and area of the apical and basal dendritic spines during hibernation regardless of tau hyperphosphorylation. The morphological changes observed here also suggest region specificity, opening up debate about a possible relationship with the differential brain activity registered in these regions in previous studies.

## Introduction

Tau is a protein that belongs to the family of the microtubule-associated proteins. Under physiological conditions, tau protein binds microtubules by tubulin interaction and participates in their assembly and stabilization, thus allowing reorganization of the cytoskeleton (Weingarten et al. 1975). In addition, tau participates in axonal transport, synaptic function, and in other novel processes beyond its habitual function as microtubule-regulating protein (Medina et al. 2016; Wang and Mandelkow 2016; Sotiropoulos et al. 2017; Ittner and Ittner 2018). The biological activity of tau is mainly regulated by post-translational modifications, particularly phosphorylation (Gong et al. 2010; Iqbal et al. 2016). Specifically, phosphorylated tau loses affinity for microtubules, promoting their destabilization, leading to cytoskeleton instability. Tau is predominantly distributed in the axon of healthy mature neurons, but, in Alzheimer’s Disease (AD) and other tauopathies, tau protein is hyperphosphorylated and, aberrantly, moves to the somatodendritic compartment where it aggregates to form paired helical filaments (PFH) (Iqbal et al. 2016). PHF are the major component of neurofibrillary tangles, which are a hallmark of AD. Several antibodies against the main phosphorylation sites have been designed to study tau hyperphosphorylation. Among them, AT8 antibody, which detects tau phosphorylation at residues Ser202 and Thr205, is an important one to highlight, as it is the most commonly antibody used to classify the degree of pathology during the development of AD (Braak and Braak 1995).

In the mammalian cerebral cortex, pyramidal cells are the most abundant neurons, estimated to represent 70–80% of the total neuronal population. They are excitatory and represent the majority of the projecting cells of the cerebral cortex. Furthermore, they constitute the major source of cortical excitatory synapses, and their dendritic spines are the principal cortical postsynaptic targets of excitatory synapses (DeFelipe and Fariñas 1992; Kanari et al. 2019). Thus, pyramidal cells are considered the main building blocks of the cerebral cortex. Differences in the patterns of dendritic branching may determine the degree to which the integration of inputs is compartmentalized within their arbors (Koch et al. 1982; Spruston 2008). Furthermore, differences in the density of dendritic spines indicate differences in the number of excitatory synaptic inputs and may also influence the local summation of postsynaptic potentials or the cooperativity between inputs (e.g., Shepherd et al. 1985; Spruston 2008). Therefore, elucidating the effect of tau phosphorylation on pyramidal neuron structure may shed light on the alterations of the integration of inputs that take place in AD.

Synapse loss has been documented in the hippocampal neurons of P301S mutant human tau transgenic mice (Yoshiyama et al. 2007). In addition, tau mislocalization to dendritic spines has been reported to cause early synaptic dysfunction by suppression of the AMPA receptor-mediated synaptic responses in a mouse model expressing P301L human tau (Hoover et al. 2010). In human AD samples, the accumulation of hyperphosphorylated tau in a pretangle state does not seem to induce changes in the dendrites of pyramidal neurons, whereas the presence of intraneuronal neurofibrillary tangles has been associated with loss of dendritic spines and dendrite atrophy, including alterations in dendritic spine head volume and dendritic spine length (Merino-Serrais et al. 2013). Importantly, tau pathology is correlated with cognitive impairment progression in AD (Nelson et al. 2012).

Hibernation is described as a period in which some winter-adapted animals save energy by entering a dormant state called torpor. In some small mammals, such as the Syrian hamster (*Mesocricetus auratus*), torpor is characterized by reduced body temperature and metabolic rate that can last for 3–4 days. Multiple bouts of torpor, interspersed with short arousal periods of activity and normothermia, occur until favorable conditions appear again (Ruf and Geiser 2015). The Syrian hamster is a facultative hibernator, which means that it may enter hibernation artificially when exposed to a short-day photoperiod and cold temperature (Chayama et al. 2016). Previous studies have demonstrated that the brain of these animals undergoes complex adaptive and reversible changes that are supposed to protect the brain from hypoxia and hypothermia. In 1992, Popov and colleagues described a retraction of dendritic trees (i.e., they became shorter and less branched) and a reduction in spine density of hippocampal CA3 pyramidal neurons during the hibernation of ground squirrels (*Spermophilus citellus*) (Popov et al. 1992). These changes were fully reversed upon emerging from hibernation (arousal). In a later study, von der Ohe observed that arborization retraction also occurred in layer IV cortical spiny stellate neurons and in thalamus and suggested a linear relationship between this retraction and the drop in body temperature (von der Ohe et al. 2006). Other similar studies revealed the loss of synaptic protein clustering and confirmed hypothermia as a trigger (Popov et al. 2007; von der Ohe et al. 2007). Moreover, Magariños et al. reported the loss of dendritic spines and reduced apical dendritic tree complexity in CA3 neurons from hibernating European hamsters (*Cricetus cricetus*). Interestingly, basal dendritic trees remained unaltered, and no variations in spine density were detected in CA1 pyramidal neurons (Magariños et al. 2006). A more recent study showed a transient spine reduction in apical dendrites of hippocampal pyramidal cells (CA1 and CA3) during the hibernation of the Syrian hamster and ruled out a memory impairment because of the seasonal and repeated neuronal changes (Bullmann et al. 2016).

Hibernation is a useful model to study tau phosphorylation and dephosphorylation events: labeling with PHF-like epitopes revealed that tau is reversibly hyperphosphorylated in torpid animal neurons (Arendt et al. 2003). In this regard, Bullmann and colleagues also proposed tau hyperphosphorylation as one of the main elements involved in the reversible synaptic regression (Bullmann et al. 2016). Thus, mammalian hibernation allows us to analyze the transient tau protein hyperphosphorylation through a natural nontransgenic animal model.

To date, there are no detailed studies of dendritic spine morphology in the neocortex of any hibernating species and the direct relationship between tau hyperphosphorylation and the morphological neuronal changes in cortical neurons needs further analysis. Here, we investigated whether tau hyperphosphorylated layer Va neocortical pyramidal neurons display any morphological alterations during the hibernation of the Syrian hamster.

## Materials and Methods

### Syrian Hamsters

A total of 14 male 4-month-old Syrian hamsters were purchased from Janvier Labs. These animals had free access to food and water and were kept at 23 °C with an 8:16 h light:dark cycle for a 4–6 week acclimatization period in our animal facility. Subsequently, as described in Antón-Fernández et al. (2015), in order to obtain the torpor experimental group, 7 of the animals were transferred to a special chamber which makes it possible to gradually reduce the temperature (via LM35 sensors), control the illumination (adjustable LED RGB), and monitor the hamsters by measuring the general locomotor activity with a PIR (passive infrared) sensor mounted on top of each cage. In addition, we recorded all data obtained in a notebook computer, distinguishing between the torpor and arousal phases during hibernation using the software package *Fastwinter* 1.9 (developed by Tiselius s.l.). Hibernating animals were considered to be torpid when they had been inactive for at least 24 h. The status of the animals was confirmed by body temperature measurements (infrared thermometer) since the body temperature of a hibernating animal falls to almost 5 °C, whereas it is about 35 °C in euthermic animals. Since torpor bouts (periods of time a hibernator spends at low body temperature) are nonregular at the start of hibernation, we considered animals torpid and ready to be sacrificed only when they had completed 3 full torpor bouts. Hamsters were sacrificed at 36–48 h of torpor—the period when the brain has been described as displaying the highest levels of hyperphosphorylated tau (Bullmann et al. 2016).

All experimental procedures were carried out at the animal facility in the San Pablo CEU University of Madrid (SVA-CEU.USP, registration number ES 28022 0000015) in accordance with the European Union Directive (2010/63/CE) and the approval of the institutional Animal Experiment Ethics Committee (No. PROEX 292/15).

### Tissue Preparation

Animals were sacrificed by a lethal intraperitoneal injection of sodium pentobarbital (200 mg/kg) and were then perfused intracardially with a saline solution followed by 4% paraformaldehyde in 0.1 M phosphate buffer (PB, pH 7.4). The brain of each animal was removed and postfixed by immersion in the same fixative for 24 h at 4 °C. Serial coronal sections (200 μm thick) were obtained with a vibratome (St Louis, MO, USA) and kept in 0.1 M PBS (phosphate buffer saline).

### DAB Immunostaining

Free-floating sections were pretreated with 1.66% H_2_O_2_ for 30 min to quench the endogenous peroxidase activity and then for 1 h in PB with 0.25% Triton-X and 3% normal goat serum (Vector Laboratories). The sections were then incubated overnight at 4 °C with a mouse antiPHF-tauAT8 antibody (Pierce Endogen, 1:2000), and the following day they were rinsed and incubated for 1 h in biotinylated goat anti-mouse IgG (1:200; Vector Laboratories). Antibody binding was detected with a Vectastain ABC immunoperoxidase kit (Vector Laboratories) and visualized with the chromogen 3,3′-diaminobenzidine tetrahydrochloride (DAB; Sigma-Aldrich). After staining, the sections were dehydrated, cleared with xylene, and covered-slipped (DePeX; Merck KGaA 100579).

### Intracellular Injections and Immunocytochemistry

Sections from both hemispheres were prelabeled with 4,6-diamidino-2-phenylindole (DAPI; Sigma), and a continuous current was used to blindly inject individual cells with Lucifer yellow (LY; 8% in 0.1; Tris buffer, pH 7.4) in layer Va of the primary sensory neocortex (area S1 according to Morin and Wood 2001). The primary somatosensory cortex was chosen in order to follow up on previously published work by our laboratory in which cellular changes were found in this cortical area during the hibernation of the Syrian hamster (microglial processes numbers increase, along with a shortening of the Iba-1 immunoreactivity; the length of the axon initial segment is significantly increased; and the Golgi apparatus of glial cells and neurons alike undergo structural modifications) (Leon-Espinosa et al. 2017; Leon-Espinosa et al. 2018; Leon-Espinosa et al. 2019). LY was applied to each injected cell by continuous current until the distal tips of each cell fluoresced brightly, indicating that the dendrites were completely filled and ensuring that the fluorescence did not diminish at a distance from the soma. 1200 pyramidal neurons of the Syrian hamsters (including both control and torpor) were injected, from which 90 cells were selected for the study based on the quality of the LY-labeled cells.

Following the intracellular injections, the sections were immunostained for LY using a rabbit antibody against LY (1:400 000; generated at the *Instituto Cajal*, Madrid) diluted in stock solution (2% bovine serum albumin, 1% Triton X-100, and 5% sucrose in PB) for 48 h. Immunostaining for LY (in stock solution) was maintained for a further 48 h, together with antiPHF-tauAT8 (mouse; 1:2000 in stock solution; MN1020, Pierce Endogen). AntiPHF-tauAT8 binding was detected with a biotinylated horse anti-mouse secondary antibody (1:200 in stock solution; BA-2000, Vector), followed by a mixture of Alexa Fluor 488 anti-rabbit (1:1000 in 0.1 M PB) and streptavidin coupled to Alexa Fluor 594 (1:1000 in 0.1 M PB; Molecular Probes). The sections were then mounted with ProLong Gold Antifade Reagent (Invitrogen Corporation) and stored at −20 °C. See (Elston et al. 2001; Benavides-Piccione and DeFelipe 2003) for further details of the cell injection method.

### Image Acquisition

Following the method described in Benavides-Piccione et al. (2013), imaging was performed with a Zeiss LSM 710 confocal microscope coupled to an Axio Observer inverted microscope (Zeiss), recording Alexa 488 (green) and 594 (red) fluorescence through separate channels. For cell reconstruction, consecutive stacks of images at high magnification (×63 glycerol; voxel size, 0.110 × 0.110 × 0.350 μm) were acquired to capture dendrites along the apical and basal dendritic arbors. For dendritic spine reconstruction, consecutive stacks of images at high magnification (×63 glycerol; voxel size: 0.057 × 0.057 × 0.140) were acquired to capture dendritic spines along the length of the main apical dendrite. It is important to note that, for each stack, the laser intensity and detector sensitivity were set such that the fluorescence signal from the dendritic spines occupied the full dynamic range of the detector. Therefore, some pixels were saturated in the dendritic shaft, but no pixels were saturated within the dendritic spines.

### Cell Reconstruction and Quantitative Analysis

The morphological analysis was performed in 3D using Neurolucida 360 (MBF Bioscience) and included 90 cells, from which 33 cells belonged to the T(AT8−) group, 27 cells belonged to the T(AT8+) group, and 30 cells belonged to the control group.

Briefly, apical and basal arbors were described through 3D points. These points have an associated diameter that provides the information of the varying thickness of the dendrite at that particular point and varies along the length of the dendrite.

Several morphological variables were extracted using Neurolucida software (see Benavides-Piccione et al. 2006 for details). As discussed in Benavides-Piccione et al. (2020), some of the features measured did not depend on the entirety of the reconstructed cell and can thus be considered as full measurements: mean soma area (estimated by measuring the area of the maximum perimeter of the soma) and average dendritic segment diameter, length, surface area, and volume. However, other morphological variables did depend on the entirety of the cell and, thus, may only partially describe the cell and can be considered “non-full” measurements: area and volume of the dendritic arbor, total number of dendrites, total number of nodes, total dendritic length, total dendritic surface area, and total dendritic volume.

Values are expressed as total numbers, per branch order segment and as a function of the distance from soma (Sholl analysis). Only dendritic segments that were completely reconstructed were included in the analysis.

### Dendritic Spine Reconstruction and Quantitative Analysis

**Figure 1:**
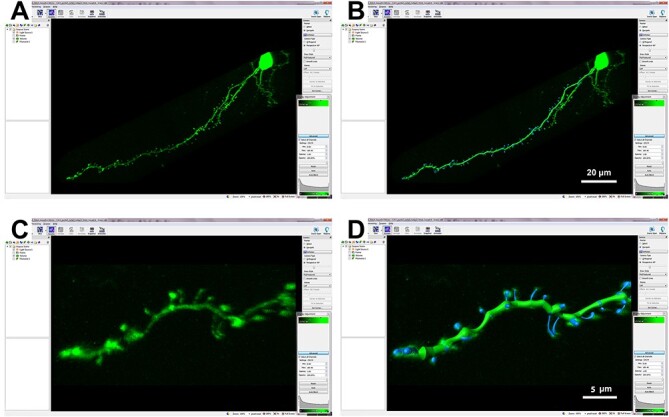
(*A*) Imaris software screenshot showing a basal dendrite belonging to a LY injected layer Va pyramidal neuron; (*B*) reconstruction of the basal dendrite shown in (*A*) using Imaris Software Filament Tracer Tool; (*C* and *D*) higher magnification of the terminal region of the dendrite shown in (*A*) and its reconstruction.

After image acquisition, the stacks were opened with three-dimensional image processing software—Imaris 7.6.4 (Bitplane AG)—and dendritic spines were individually reconstructed using Filament Tracer Tool in the main apical dendrite (*n* = 31 cells in control group; *n* = 33 cells in T(AT8−) group; *n* = 26 cells in T(AT8+) group) and in randomly selected basal dendrites (*n* = 25 cells in all groups) that run parallel to the cortical surface. The dendritic spine density was established as the number of dendritic spines found in segments of 10 μm along the length of the dendrite. Dendritic spine length, area, and volume were obtained using the same software (Fig. 1).

### Statistical Analysis

All statistical analyses were performed using GraphPad Prism version 5.00 for Windows (GraphPad Software). When morphological parameters were presented as mean values, the Kruskal–Wallis test was used to compare between the groups. Measurements reported as a function of the distance from the soma were analyzed using a two-way ANOVA test. Differences were considered to be significant when *P* < 0.05. Measurements are reported as mean ± SEM, unless otherwise indicated.

### Methodological Considerations

Since the intracellular injections of the pyramidal cells were performed in 200-μm-thick coronal sections, the part of the dendritic arbor nearest the surface of the slice from which the cell soma was injected (typically at a depth of ∼30 μm from the surface) was lost. It is important to note that the percentage of the basal arbor and apical arbor included within the section may vary in each cell depending on how parallel the main apical dendrite runs with respect to the surface of the slice. In the present study, neurons were included in the analysis if they had a main apical dendrite length of at least 200 microns. Furthermore, dendrites that ran for further than ∼900 μm from the soma were not properly filled with dye, and, therefore, distal apical dendrites (apical tufts) of layer Va cells were not included in the analysis.

In addition, as hyperphosphorylated tau was mainly observed in the proximal region of the apical dendrite emerging from the soma, we limited the study of the apical arbor to the first 180 μm from the soma. Thus, the changes described here during hibernation may not necessarily reflect the alterations in the remaining cell structure. Due to technical limitations (mostly incomplete filling of the cells by LY), tridimensional spine analysis was performed in the proximal regions of the apical dendrite, since distal apical dendrites (e.g., apical tufts) could not be included in the analysis.

## Results

### Different Tau Hyperphosphorylation (AT8) Patterns in Pyramidal Neurons from Layer V

**Figure 2:**
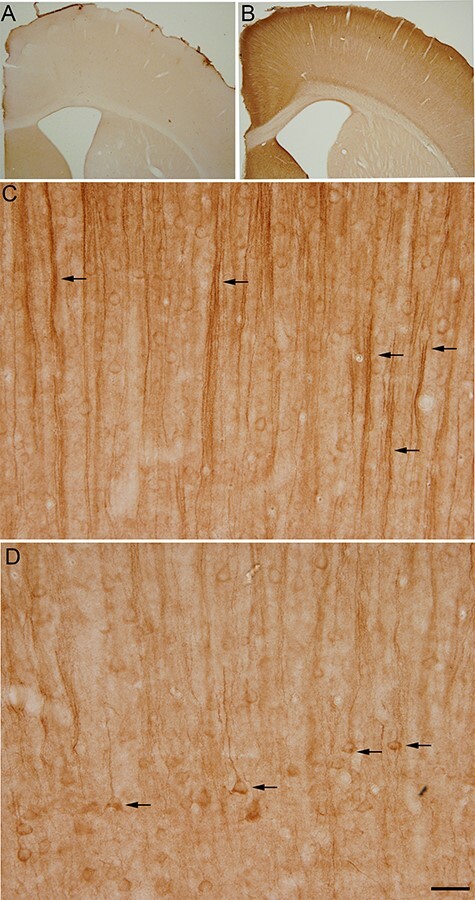
Photomicrographs showing the patterns of hyperphosphorylated tau immunostaining (using AT8 antibody) in the brain of control (*A*) and torpid (*B*) Syrian hamsters. Representative images from layer II–III (*C*) and layer IV–V (*D*) somatosensory cortical layers are shown. Arrows in *C* point out apical dendrites from lower layer cells. Arrows in *D* indicate some AT8-positive layer Va neurons. Scale bar in *D* indicates 750 μm in (*A*,*B*) and 75 μm in (*C*,*D*).

**Figure 3:**
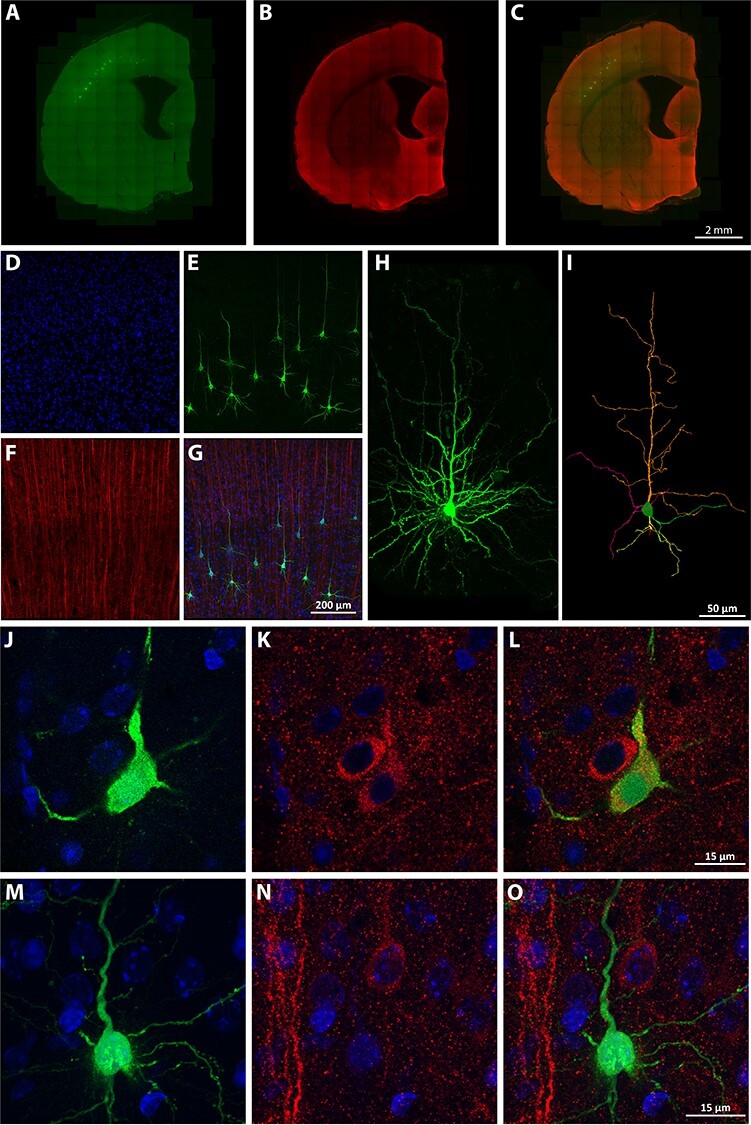
(*A*–*C*) Representative panoramic images of pyramidal neurons injected with LY in the somatosensory cortex; LY injections are shown in *A*, AT8 immunostaining in *B*, and they are merged in *C*. (*D*–*G*) Higher magnification confocal image of a group of injected pyramidal neurons showing DAPI staining (*D*), LY staining (*E*), AT8 staining (*F*), and *D*–*F* merged (*G*). (*H*) Higher magnification photomicrograph of a representative injected pyramidal neuron. (*I*) 3D reconstruction of the same cell. (*J*–*O*) High magnification confocal microscopy images of 2 different injected somatosensory layer V-injected cells, showing an injected pyramidal neuron with positive PHF-tauAT8 immunostaining (and therefore included in the T(AT8+) group (*J*–*L*)) and an injected pyramidal neuron showing negative PHF-tauAT8 immunostaining (and therefore included in the T(AT8−) group (*M*–*O*)). For all images: DAPI is shown in blue, LY is shown in green, and AT8 is shown in red. Scale bars are shown at the bottom of each set of images: 2 mm in *A*–*C*, 200 μm in *D*–*G*, 50 μm in *H*–*I*, and 15 μm in *J*–*O*.

As previously described, AT8 immunostaining showed that tau hyperphosphorylation occurs upon hibernation and that it follows a nonhomogenous pattern (Arendt et al. 2003). In this study, we chose the subpopulation of pyramidal cells located in layer Va because some of those neurons displayed a strong phospho-tau expression (T(AT8+)), whereas other neighboring pyramidal neurons were lightly labeled or not labeled (Fig. 2). Thus, these T(AT8+) neurons were suitable to analyze the possible effects of tau phosphorylation on cortical pyramidal cell morphology by making comparisons with neighbor non-tau phosphorylated cells (T(AT8−)). The accumulation of hyperphosphorylated tau in layer Va pyramidal neurons was restricted to the soma and the main apical dendritic shaft that emerges from the soma. AT8 labeling was not detected in the basal arbor, in the axon, or within the dendritic spines. However, the dendrites that ascend through superficial layers from lower cortical layers also displayed an evident and strong AT8 labeling (see Figs 2*C* and 3*F*,*G*).

### Neuronal Tree Reconstruction

To analyze the effect of tau phosphorylation on dendritic and spine morphology, we performed Lucifer yellow intracellular injections in the somatosensory cortex (Fig. 3). To study a representative cohort of cells, we injected specifically the Va layer cells of control (nonhibernating) and torpor animals, where cells with high AT8 immunoreactivity are relatively abundant (Fig. 3 D-G). The injected cells from torpid animals (60 cells) were divided into 2 different groups depending on the presence or absence of PHF-tauAT8 labeling: 1) T(AT8−) (33 cells; see Fig. 3*M*–*O*), which did not have any detectable AT8 labeling and 2) T(AT8+) (27 cells; see Fig. 3*J*–*L*), which displayed prominent AT8 labeling in the soma and the apical dendrite. It is important to note that, in some injected cells, AT8 labeling was present at the distal region of the apical dendrite but not in the soma and proximal regions. The number of cells following this pattern was difficult to estimate due to technical issues, such as the inclination of the slice, the penetration of the antibodies used, or the quality of the intracellular injections. These cells were not selected for the study, thus restricting the analysis to cells with positive AT8 labeling in the soma, as they are interesting for the characterization of the phosphorylated tau distribution within neurons.

**Figure 4:**
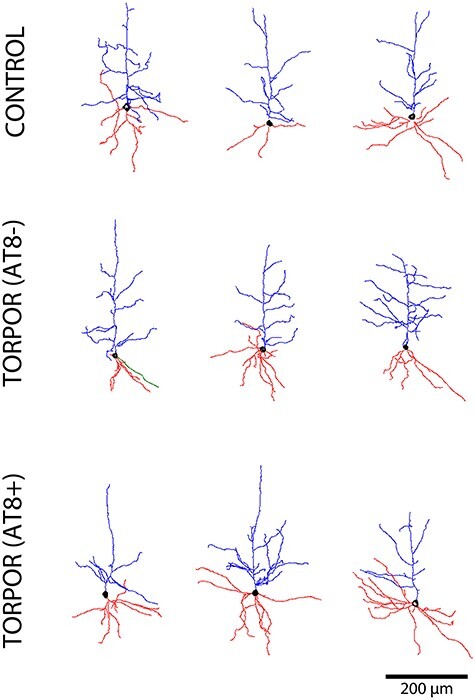
Drawings of the apical (blue) and basal (red) dendritic arbors of 3 representative somatosensory layer Va pyramidal neurons belonging to the control, T(AT8−), and T(AT8+) groups. Axons are shown in green. Scale bar, 200 μm.

The dendritic fields of the cells were reconstructed through manually traced 3D points, and the data points of neuron morphology of each pyramidal cell were included in the comparative analysis between groups (Fig. 4).

Both apical and basal arbors were analyzed separately regarding the number of intersections, number of endings, number of nodes, average diameter, length, surface area and volume. Each measurement was expressed as a function of the distance from soma (Sholl analysis) and as a total average value.

### Apical Arbor

#### Numbers of Intersections, Nodes, and Endings in Apical Arbor Lower during Hibernation

**Figure 5:**
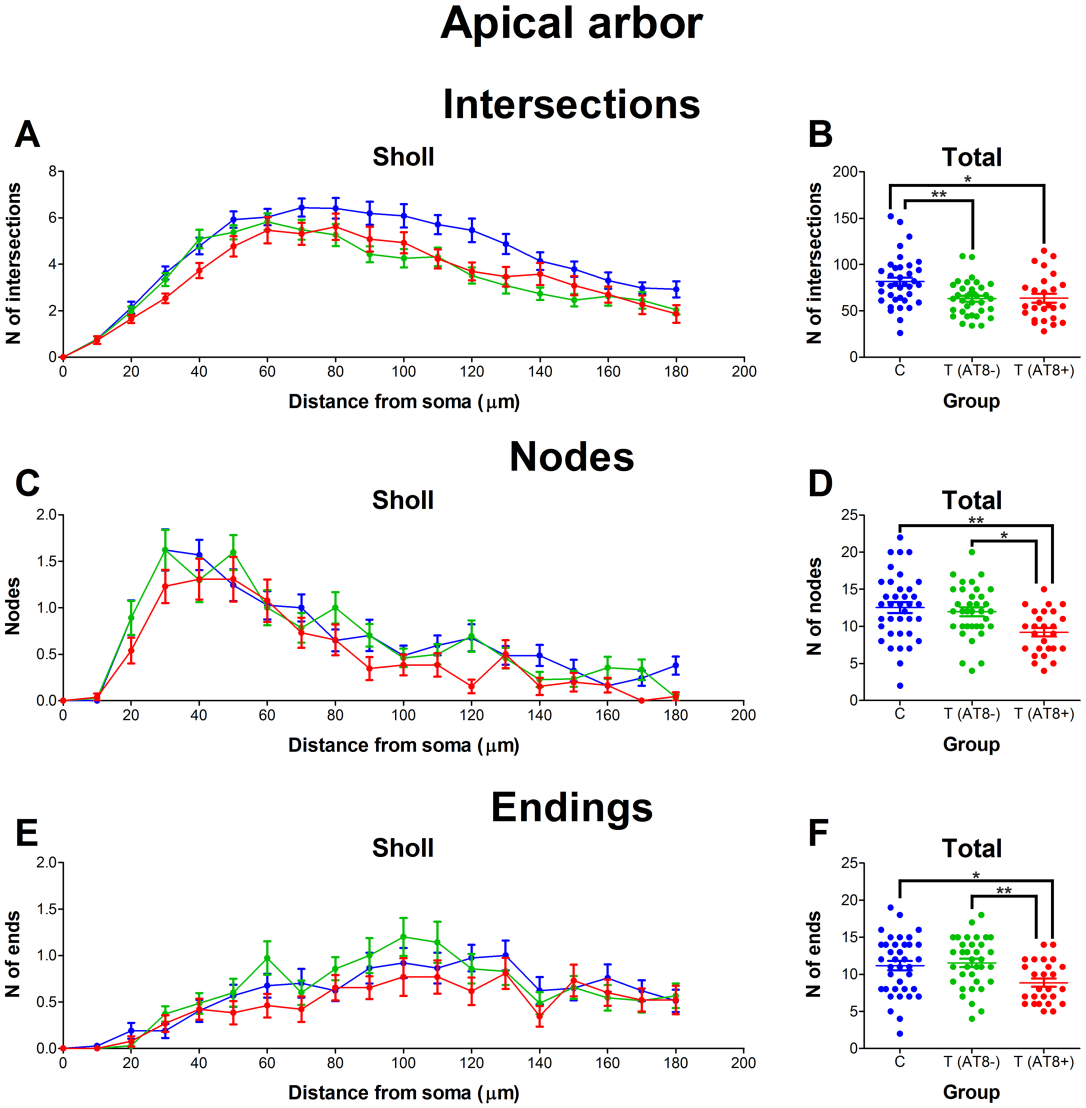
Graphs showing some morphological features of the apical dendritic arbor: apical arbor intersections (*A*, *B*), nodes (*C*, *D*) and endings (*E*, *F*), expressed as a function of the distance from soma (Sholl analysis) and as total average values.

Regarding Sholl analysis, the torpor group (both T(AT8−) and T(AT8+)) had significantly fewer intersections than the control group. Control neurons had significantly higher values at 90, 100, 120, and 130 μm from soma when compared to T(AT8−) and at 120 μm from soma when compared to T(AT8+) (Fig. 5*A*; Supplementary Tables 1 and 2). The number of nodes and the number of endings were significantly reduced in the T(AT8+) group when compared to the control and T(AT8−) groups. Again, all groups followed the same pattern in the Sholl graph, but T(AT8+) cells had lower values (Fig. 5*B*,*C*; Supplementary Tables 3–5).

#### No Variations in Mean Values for Diameter, Length, Surface Area, or Volume of the Apical Arbor during Hibernation

**Figure 6:**
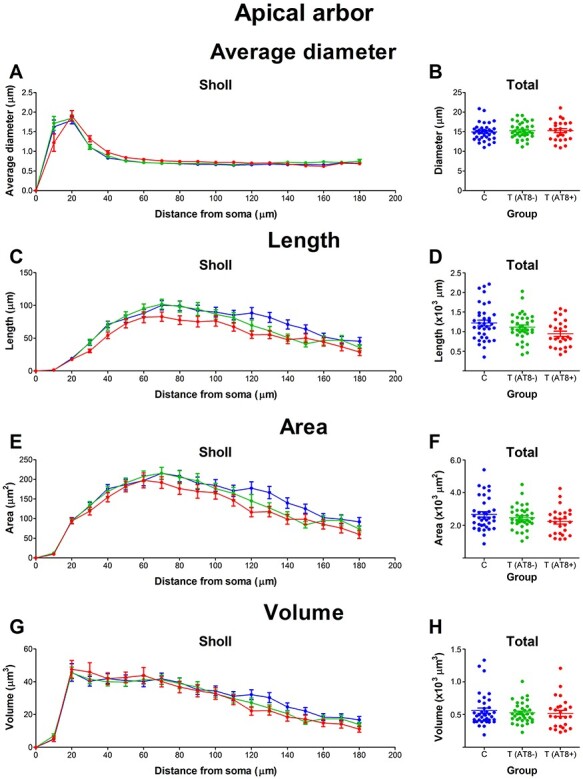
Graphs showing some morphological features of the apical dendritic arbor: apical arbor average diameter (*A*, *B*), length (*C*, *D*), surface area (*E*, *F*), and volume (*G*, *H*), expressed as a function of the distance from soma (Sholl analysis) and as total average values.

In terms of average dendritic diameter, no significant differences between groups were found when total values were compared (Fig. 6*A*,*B*). However, there was a significantly lower dendritic diameter in the T(AT8+) group at a distance of 10 μm from soma (Fig. 6*A*; Supplementary Table 6). Interestingly, the proximal areas were the ones that displayed the highest hyperphosphorylated tau. Regarding total values of dendritic length, surface area, and volume, no significant differences were found between groups (Fig. 6*B*,*D*,*F*,*H*). However, Sholl graphs did have lower values in the torpor group, mostly in the T(AT8+), with significantly lower length and surface area values at 120 μm from soma compared to control (Fig. 6*C*,*E*,*G*; Supplementary Tables 7 and 8).

In summary, when comparing the first 180 μm Sholl distance of the apical arbor from torpor and control layer Va neurons, significant differences appear in terms of total number of intersections, nodes, and endings. Regarding nodes, both torpor groups, T(AT8−) and T(AT8+), had fewer total nodes than the control group, whereas the total number of intersections and total number of endings were lower than control only in the case of the T(AT8+) group. Regarding length, area, and volume, no significant differences were found in the total values, but Sholl graphs did show a tendency for the T(AT8+) group values being lower than those of the control and T(AT8−) groups.

To further characterize the morphology of apical arbors, we analyzed the distance from soma at which oblique branches emerge. To do so, we calculated the distance at which the nodes of the main apical dendrite were located. No significant differences between groups were found up to the sixth oblique branch (Supplementary Fig. 1). Thus, hibernation does not affect the distance at which first oblique branches emerge from the apical arbor.

### Basal Arbor

#### No Variations in Mean Values for Intersections, Nodes, and Endings in the Basal Arbor during Hibernation

**Figure 7:**
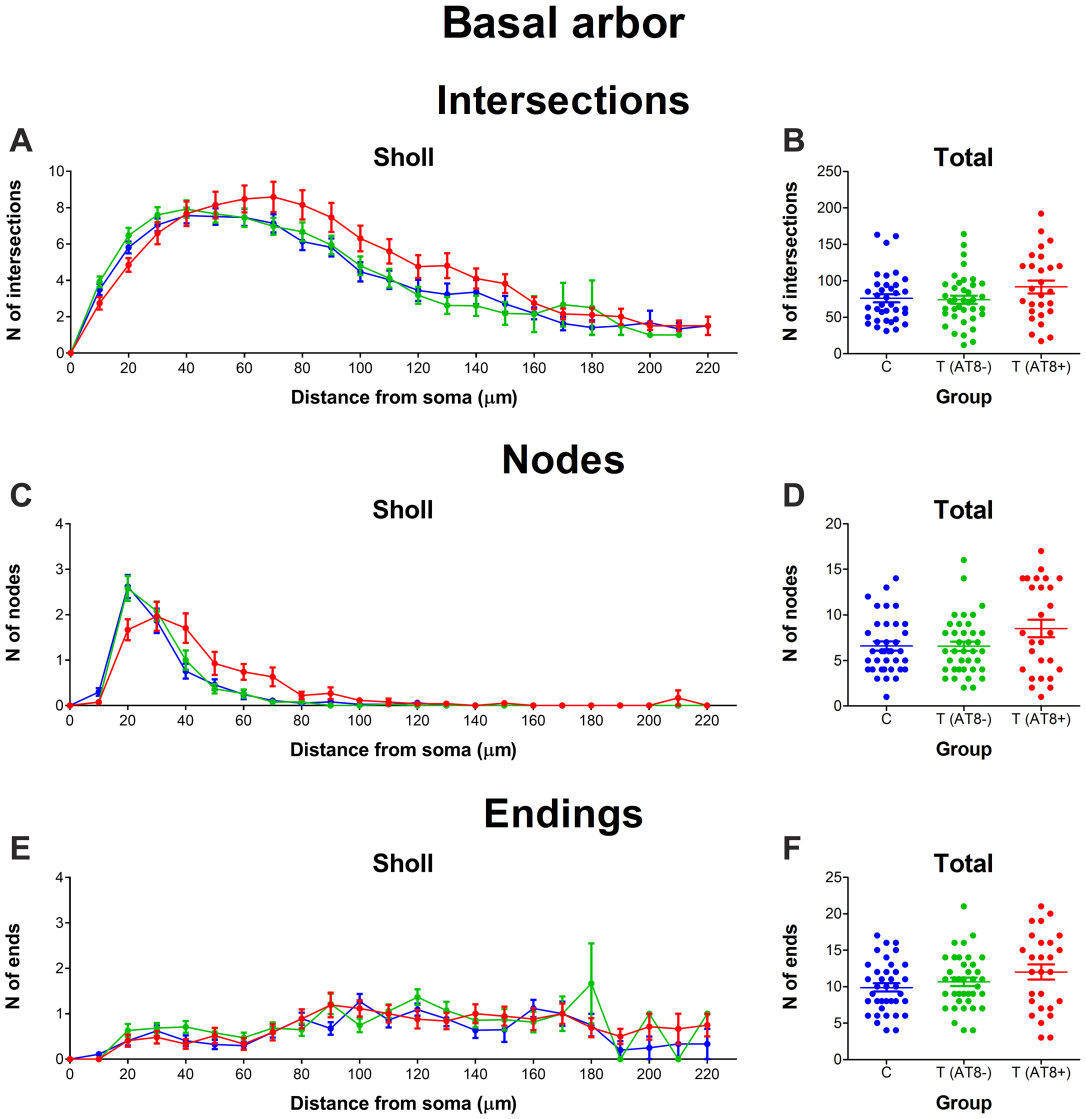
Graphs showing some morphological features of the basal dendritic arbor, including basal arbor intersections (*A*, *B*), nodes (*C*, *D*), and endings (*E*, *F*), expressed as a function of the distance from soma (Sholl analysis) and as total average values.

Regarding the number of intersections, nodes, and endings, no significant differences between groups were found when comparing mean total values (Fig. 7*B*,*D*,*F*). However, the analysis of the number of nodes based on the distance from soma (Sholl analysis) revealed significantly higher values at 20 and 40 μm for the T(AT8+) group compared to both the control group and the T(AT8−) group (Fig. 7*C*; Supplementary Table 9).

#### Basal Arbor Diameter is Higher in the T(AT8+) Group, Whereas Length, Surface Area, and Volume Mean Values Remain Constant during Hibernation

**Figure 8:**
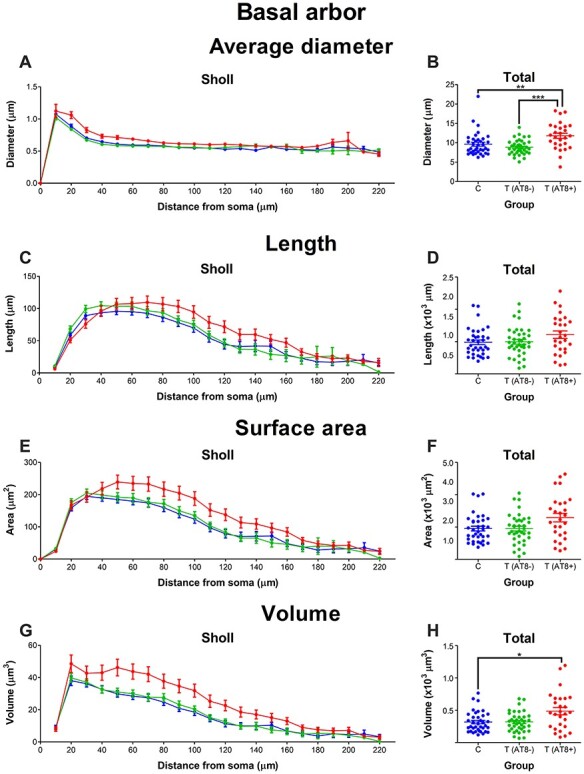
Graphs showing some morphological features of the basal dendritic arbor, including basal arbor average diameter (*A*, *B*), length (*C*, *D*), surface area (*E*, *F*), and volume (*G*, *H*), expressed as a function of the distance from soma (Sholl analysis) and as total average values. ^*^P < 0.05; ^**^P < 0.001; ^***^P < 0.0001.

Regarding average dendritic diameter, the T(AT8+) group had significantly higher total mean values than the T(AT8−) and the control groups (Fig. 8*B*; Supplementary Table 11). The Sholl curve also reflected this, with significantly higher values at 20 and 30 μm from soma when compared to the control group and 20–60 μm from soma when compared to the T(AT8−) group (Fig. 8*A*; Supplementary Table 10). Considering dendritic length, no significant differences were found when comparing total length values or comparing between Sholl curves (Fig. 8*C*,*D*). In addition, no significant differences were found for total surface area comparisons; however, unlike with length, in the case of these 2 variables, significant differences were apparent from the Sholl graphs. For surface area, the T(AT8+) values were significantly higher at 90 and 100 μm from soma when compared to the control group but not compared to T(AT8−) (Fig. 8*E*; Supplementary Table 12). In the case of volume, T(AT8+) mean total values were significantly higher than control and were significantly higher from 50 to 100 μm from soma when compared to the control group and from 50 to 70 μm from soma when compared to the T(AT8−) group (Fig. 8*G*,*H*; Supplementary Tables 13 and 14).

To sum up, when comparing basal dendrites, the average diameter was significantly higher in the T(AT8+) group than in the control and T(AT8−) groups. Moreover, Sholl graphs showed a tendency of higher values in T(AT8+) compared to the resting groups for all variables analyzed.

### Dendritic Segment Analysis

Segments were classified into branching segments (a segment that bifurcates) and terminal segments (a segment that ends).

#### Apical Arbor

**Figure 9:**
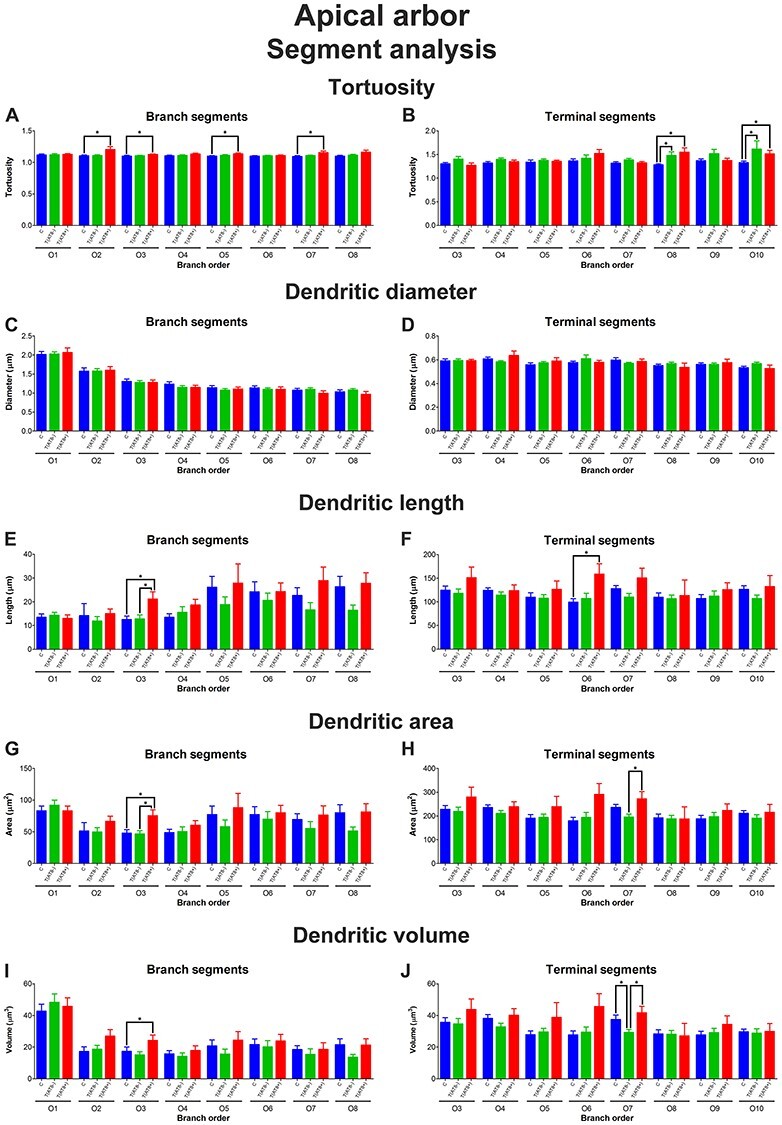
Graphs showing the segment analysis for the apical dendrites. For the 3 groups, C (blue), T(AT8−) (green), and T(AT8+) (red), the following variables are shown: tortuosity, for branch segments (*A*) and terminal segments (*B*); diameter, for branch segments (*C*) and terminal segments (*D*); length, for branch segments (*E*) and terminal segments (*F*); area, for branch segments (*G*) and terminal segments (*H*); and volume, for branch segments (*I*) and terminal segments (*J*). Mean ± SD. ^*^P < 0.05.

Our results regarding the apical arbor indicate that tortuosity (Fig. 9*A*,*B*; Supplementary Tables 15 and 19)—along with the length (Fig. 9*E*,*F*; Supplementary Tables 16 and 20), the area (Fig. 9*G*,*H*; Supplementary Tables 17 and 21), and volume (Fig. 9*I*,*J*; Supplementary Tables 18 and 22) of dendritic segments that composed the apical arbor—was higher in torpor in comparison with the control and T(AT8−) experimental groups, both regarding terminal and branching segments; the differences were statistically significant at certain specific branch orders. However, we did not perceive any significant difference in the segment diameter, regardless of the segment type or branch order (Fig. 9*C*,*D*).

#### Basal Arbor

**Figure 10:**
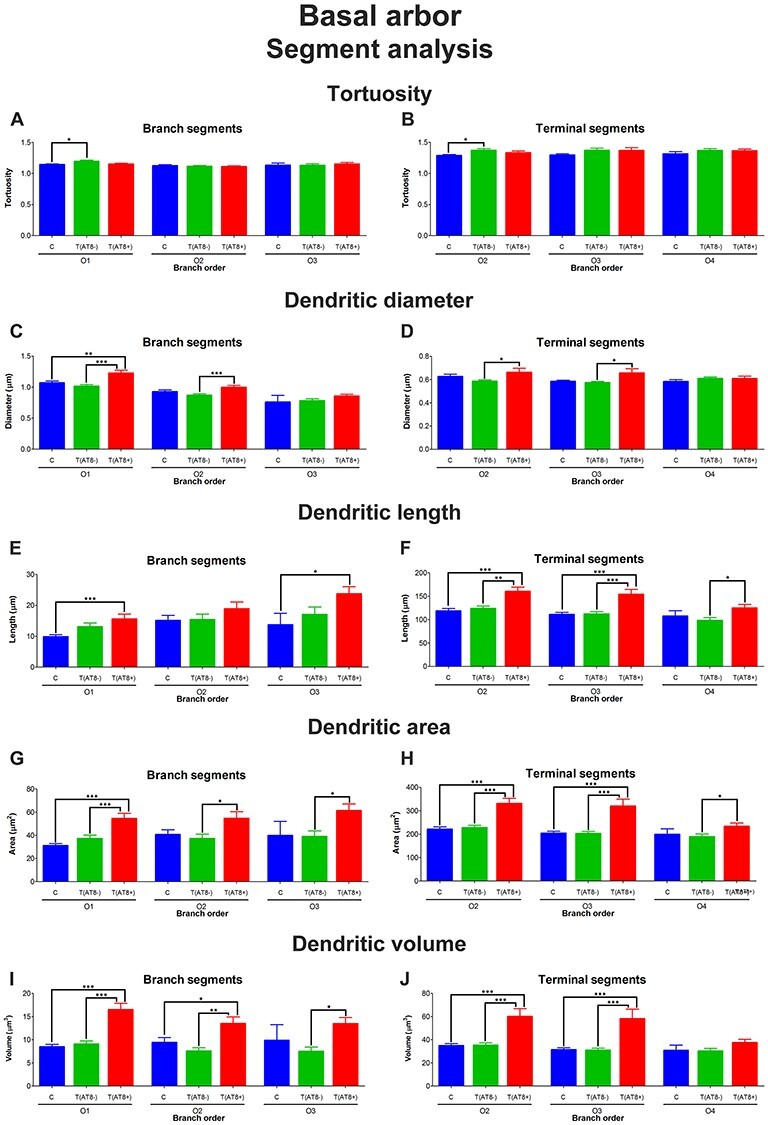
Graphs showing the segment analysis for the apical dendrites. For the 3 groups, C (blue), T(AT8−) (green), and T(AT8+) (red), the following variables are shown: tortuosity, for branch segments (*A*) and terminal segments (*B*); diameter, for branch segments (*C*) and terminal segments (*D*); length, for branch segments (*E*) and terminal segments (*F*); area, for branch segments (*G*) and terminal segments (*H*); and volume, for branch segments (*I*) and terminal segments (*J*). Mean ± SD. ^*^P < 0.05; ^**^P < 0.001; ^***^P < 0.0001.

When the basal tree segments were analyzed, we observed higher values for their length, area, and diameter in the case of T(AT8+) for terminal and branching segments at specific branch orders (Fig. 10*C*–*J*; Supplementary Tables 24–27 and 28–31), but no significant differences were observed regarding tortuosity in this group (Fig. 10). However, a significant increase was found for tortuosity in T(AT8−) group both in branching and terminal segments (Fig. 10*A*,*B*; Supplementary Tables 23 and 27).

### Dendritic Spine Reconstructions

Complete reconstructions were carried out for the dendritic spines in the main apical dendrite and in some basal dendrites. Several morphological values, as well as dendritic spine density, were analyzed by making comparisons with non-tau phosphorylated nearby cells or with control cells from non-hibernating animals.

#### Dendritic Spine Density during the Hibernation of the Syrian Hamster

**Figure 11:**
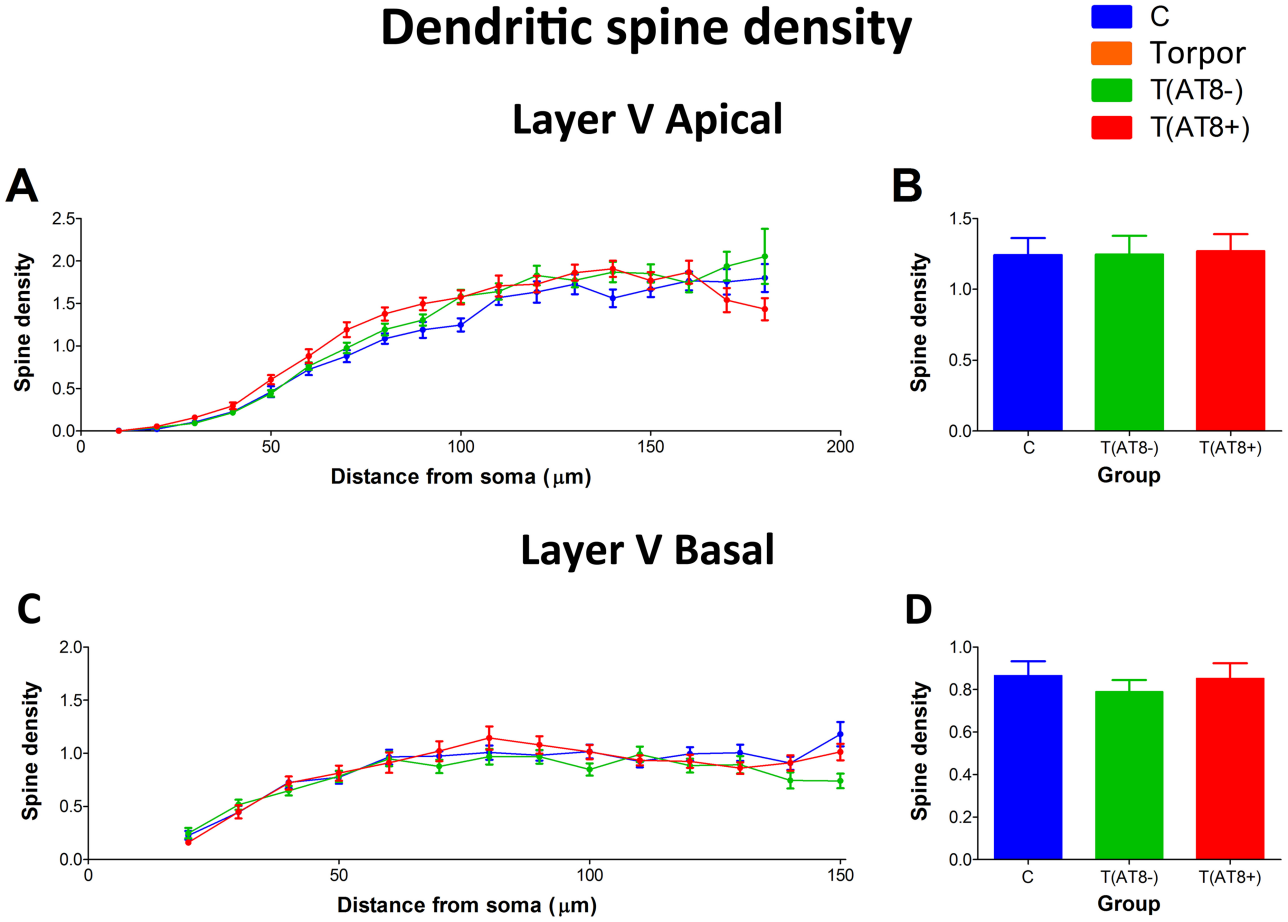
Graphs showing the dendritic spine density (per µm) in the apical main dendrite (*A*, *B*) and basal dendrites (*C*, *D*) of cortical layer V pyramidal neurons expressed as a function of the distance from soma (*A*, *C*) and as total average values (*B*, *D*).

Dendritic spine density in the proximal apical dendrite was not significantly altered between groups, except at 100 μm from soma between C and T(AT8−) groups (Fig. 11*A*,*B*; Supplementary Table 32). Regarding basal dendrites, dendritic spine density values remained constant between groups. This can be observed when data are shown as a function of the distance from soma, as total values, and as a frequency distribution (Fig. 11*C*,*D*). Worth noting that preliminary results in our laboratory indicated that the dendritic spine density is lower in CA3 cells. We examined collateral branches of apical dendrites of pyramidal cells and found that the density of spines was 3.145 ± 0.156 spines/µm (mean ± sd) in control animals (n = 6 dendrites), whereas in torpor animals was 2.724 ± 0.132 spines/µm (n = 6 dendrites). This result confirms previous observations about spine density in the hippocampal CA3 neurons upon hibernation (Popov et al. 1992; Magariños et al. 2006; Bullmann et al. 2016).

#### Spine Morphology in Apical Main Dendrite

**Figure 12:**
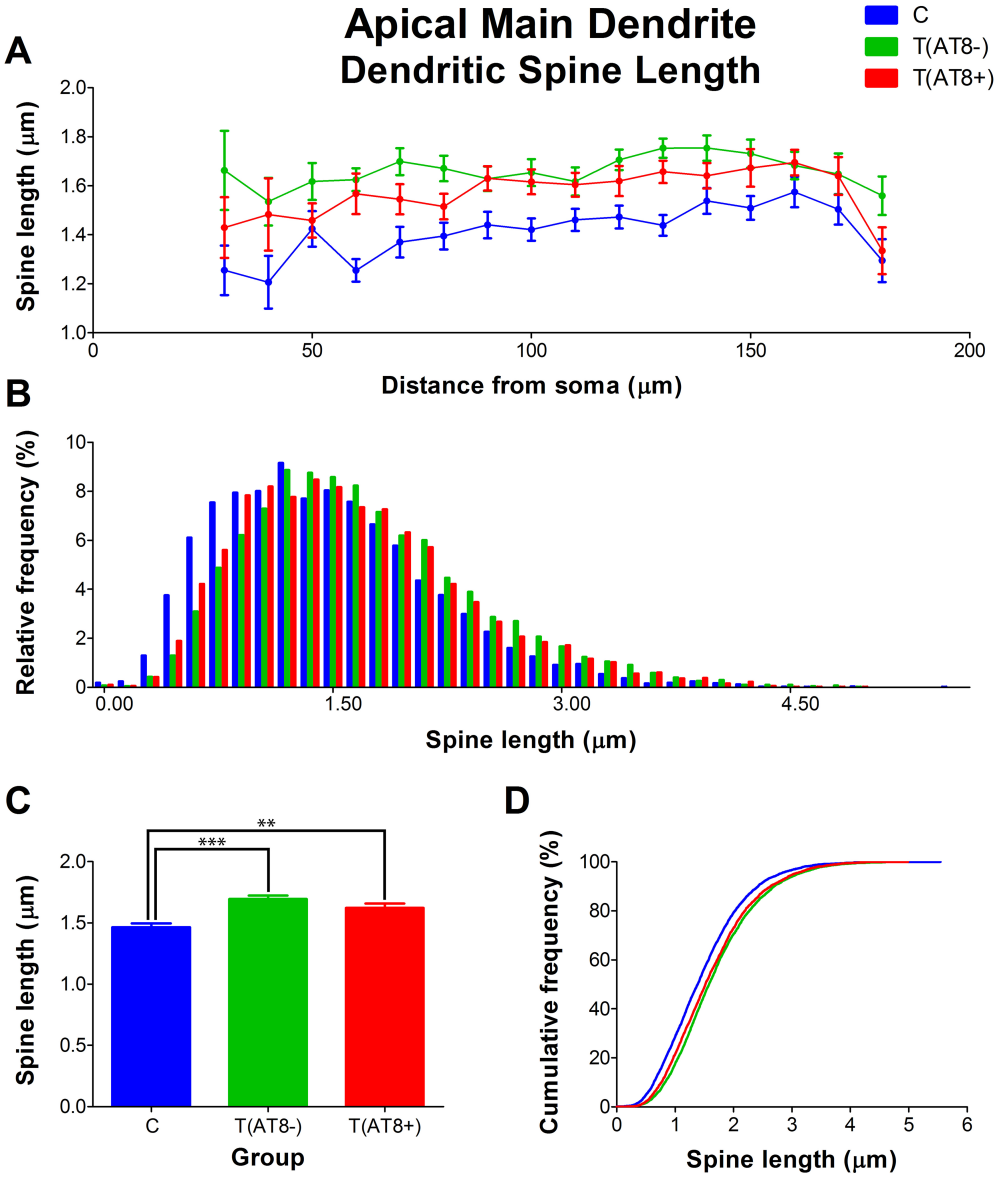
Graphs showing hamster somatosensory pyramidal layer V neuron dendritic spine length of the main apical dendrite, expressed as a function of the distance from soma (Sholl analysis) (*A*), as a relative frequency distribution (*B*), as total average values (*C*), and as a cumulative frequency distribution (*D*). ^**^P < 0.001; ^***^P < 0.0001.

**Figure 13:**
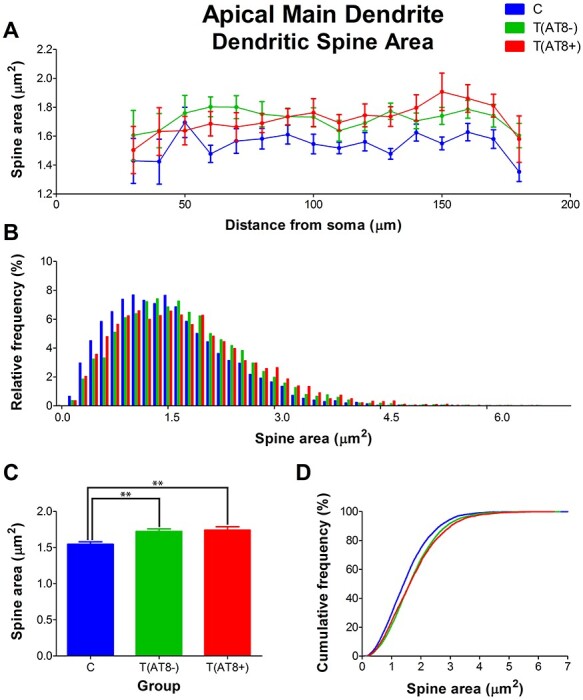
Graphs showing the dendritic spine area in the main apical dendrite, expressed as a function of the distance from soma (Sholl analysis) (*A*), as a relative frequency distribution (*B*), as total average values (*C*), and as a cumulative frequency distribution (*D*). ^**^P < 0.001.

Significant differences were found in the morphology of dendritic spines when comparing average spine length, with higher values in the T(AT8+) and T(AT8−) groups compared to the control group. The Sholl graph also reflected this difference, with the control curve appearing below the T(AT8+) and T(AT8−) curves. Moreover, data represented as a frequency distribution had larger frequencies in small spines for the control group than in the torpor groups (Fig. 12; Supplementary Tables 33 and 38). Similarly, when we compared dendritic spine area, the control group had significantly smaller spines than the torpor groups (Fig. 13; Supplementary Table 39).

**Figure 14:**
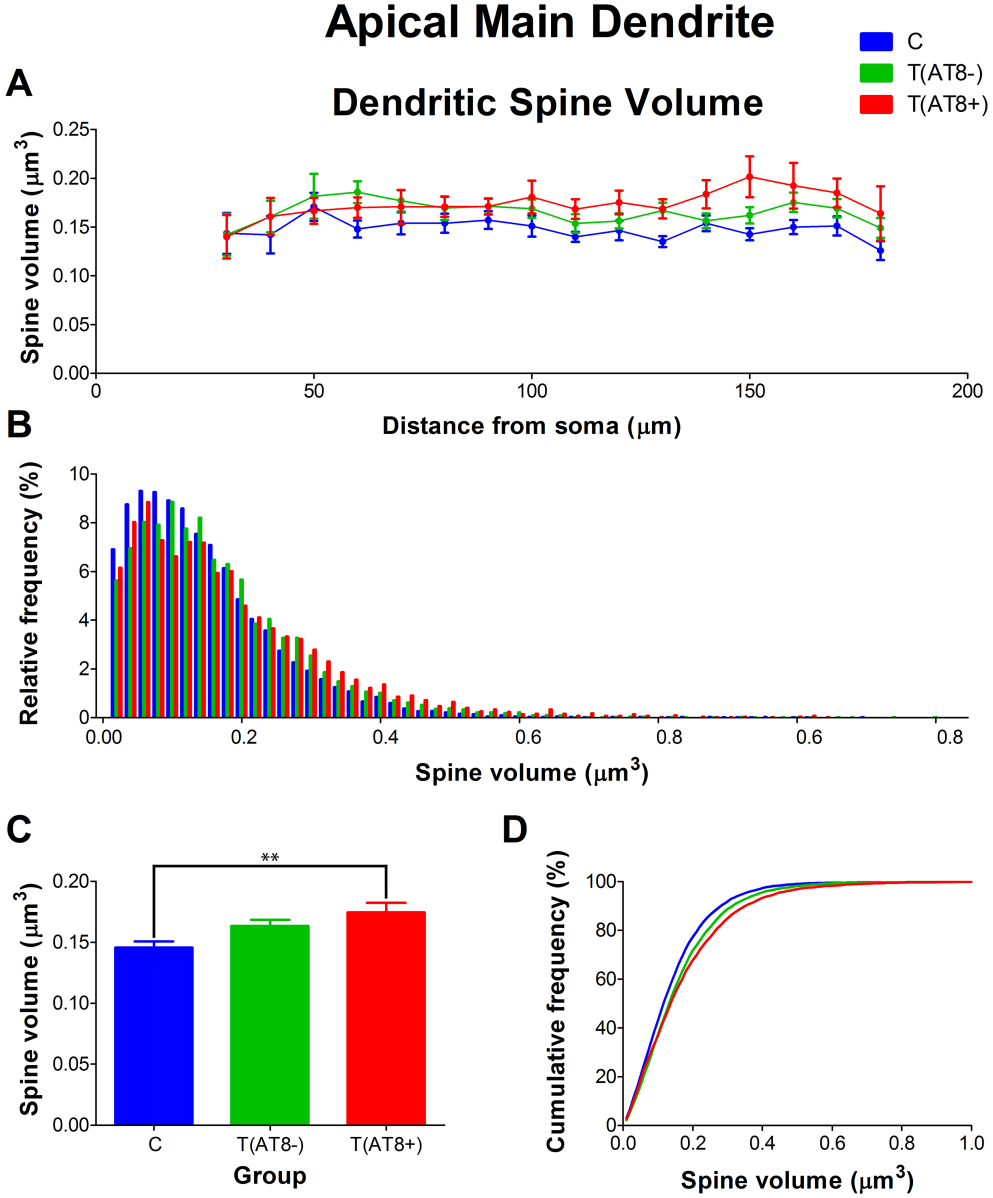
Graphs showing the dendritic spine volume in the main apical dendrite, expressed as a function of the distance from soma (Sholl analysis) (*A*), as a relative frequency distribution (*B*), as total average values (*C*), and as a cumulative frequency distribution (*D*). ^**^P < 0.001.

Finally, dendritic spine volumes for the T(AT8+) and T(AT8−) groups were significantly bigger than those from the control group (Fig. 14; Supplementary Tables 34 and 40).

To sum up, no significant differences in spine density values were found between groups. However, in the torpor group, the spines were significantly longer and larger on average compared to control.

#### Spine Morphology in Basal Dendrites

**Figure 15:**
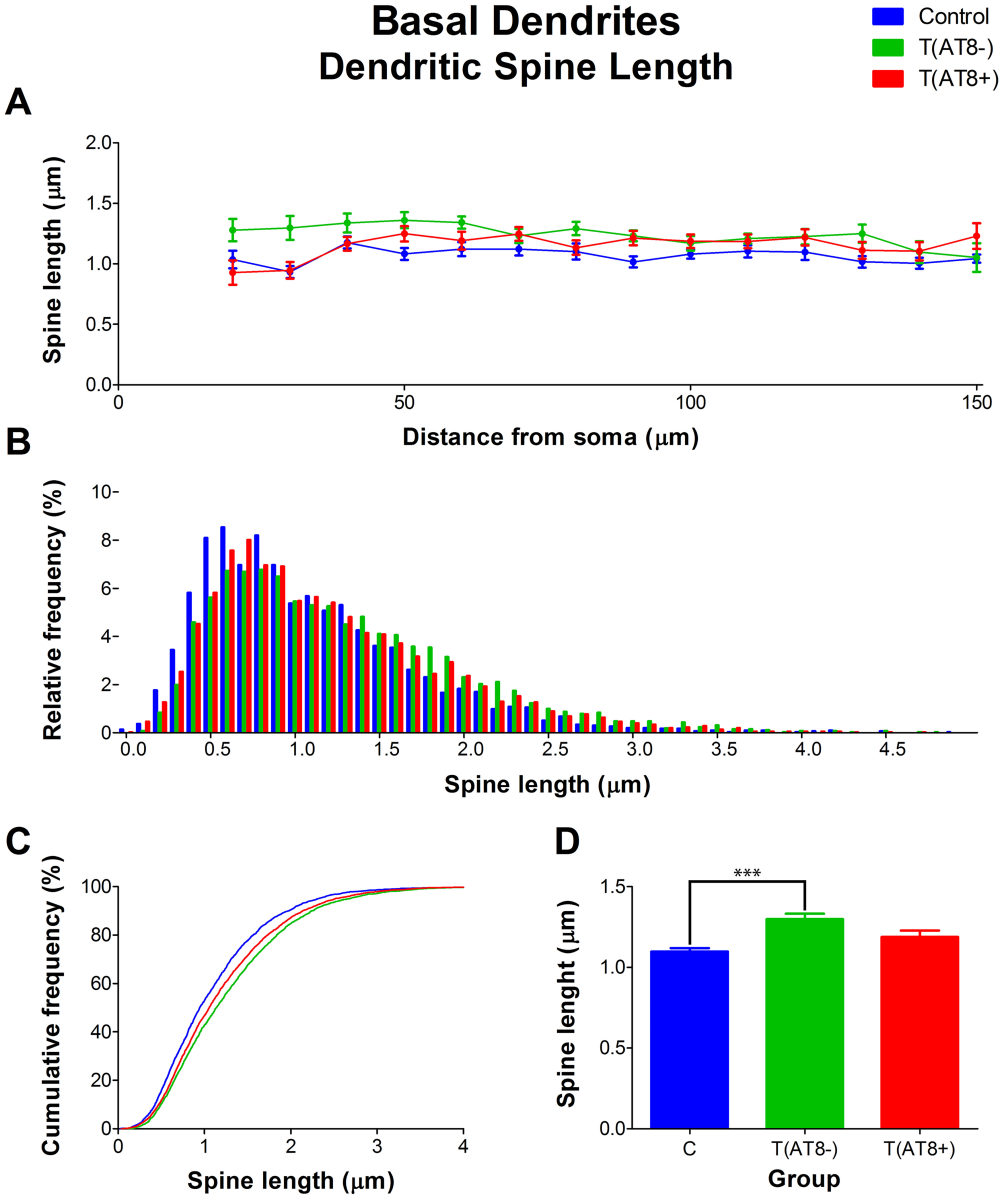
Graphs showing hamster somatosensory pyramidal layer V neuron dendritic spine length of the basal dendrites, expressed as a function of the distance from soma (Sholl analysis) (*A*), as a relative frequency distribution (*B*), as total average values (*C*), and as a cumulative frequency distribution (*D*). ^***^P < 0.0001.

Regarding the morphology of dendritic spines in the basal dendrites, a significantly higher dendritic spine length was found in T(AT8−) cells compared to control cells. However, no significant differences were found between the T(AT8+) group and the control group (Fig. 15; Supplementary Tables 35 and 41).

**Figure 16:**
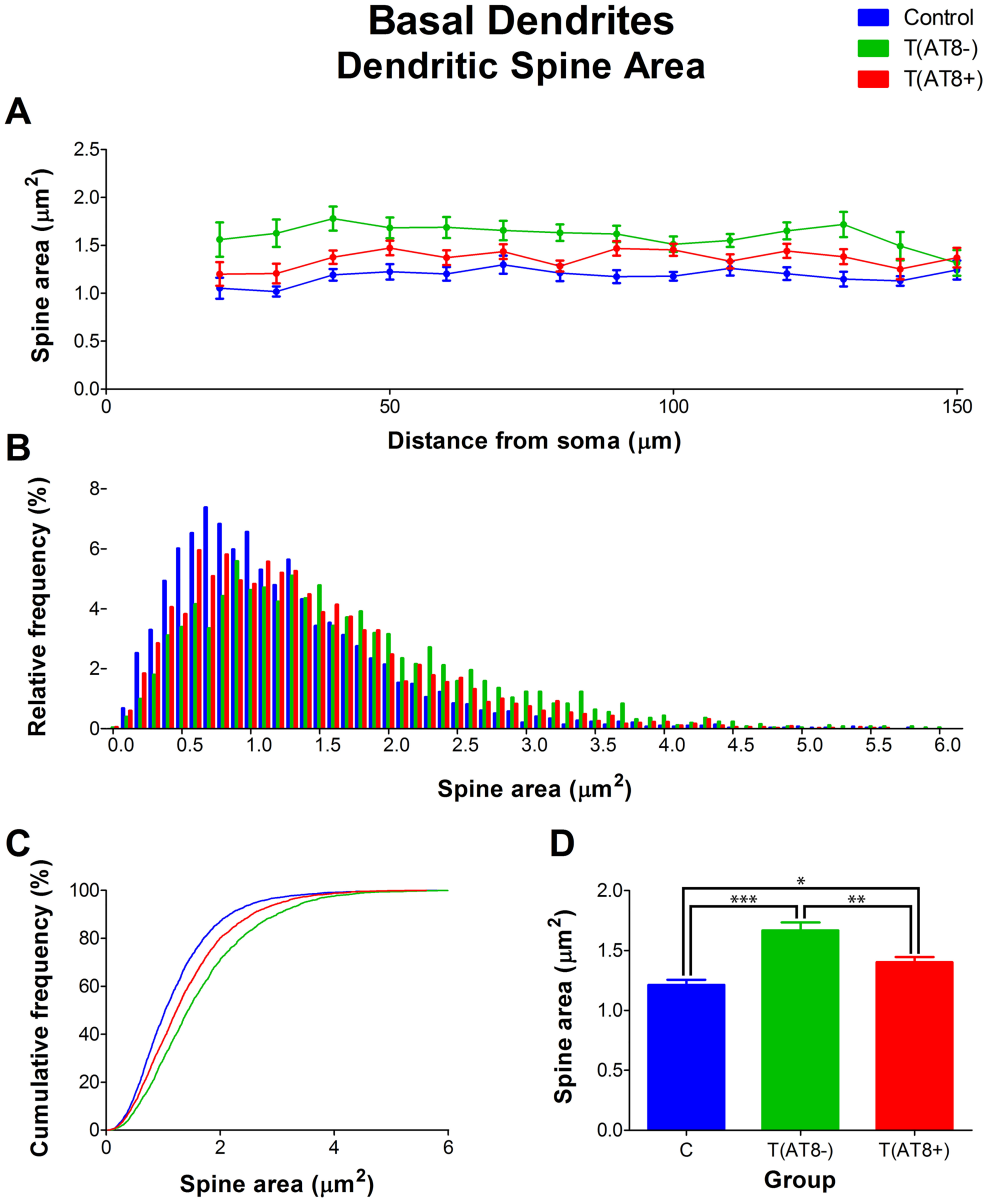
Graphs showing hamster somatosensory pyramidal layer V neuron dendritic spine area of the basal dendrites, expressed as a function of the distance from soma (Sholl analysis) (*A*), as a relative frequency distribution (*B*), as total average values (*C*), and as a cumulative frequency distribution (*D*). ^*^P < 0.05; ^**^P < 0.001; ^***^P < 0.0001.

Moreover, dendritic spine area was significantly higher in both the T(AT8−) and T(AT8+) groups compared to the control group. Moreover, the T(AT8−) group had significantly higher values than the T(AT8+) group (Fig. 16; Supplementary Tables 36 and 42).

**Figure 17:**
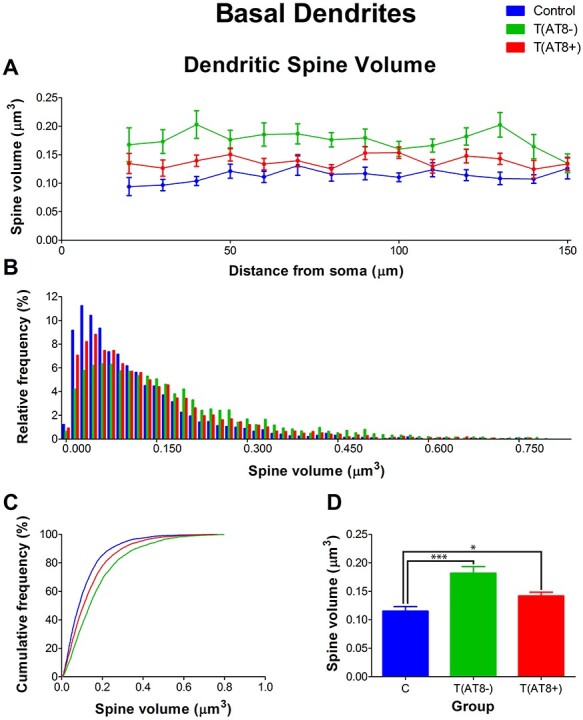
Graphs showing hamster somatosensory pyramidal layer V neuron dendritic spine volume of the basal dendrites, expressed as a function of the distance from soma (Sholl analysis) (*A*), as a relative frequency distribution (*B*), as total average values (*C*), and as a cumulative frequency distribution (*D*). ^*^P < 0.05; ^***^P < 0.0001.

Furthermore, regarding dendritic spine volume, values were significantly higher in both the T(AT8−) and T(AT8+) groups compared to the control group (Fig. 17; Supplementary Tables 37 and 43).

Finally, cell body area was analyzed, and no significant differences were found between groups, with area values of 144.5 ± 30.20 μm^2^ in the control group, 139.4 ± 25.54 μm^2^ in the T(AT8−) group, and 162.6 ± 42.27 μm^2^ in the T(AT8+) group. A summary of all the results obtained for the apical dendrites are shown in Tables 1–3—for apical dendrites, basal dendrites, and dendritic spine morphology, respectively.

**Table 1:** Summary of the analysis regarding differences observed during hibernation of the Syrian hamster concerning apical dendrites

			C vs. T(AT8−)	C vs. T(AT8+)	T(AT8−) vs. (AT8+)
Apical arbor Whole-cell reconstructions	Intersections	Total	**	^*^	ns
		Sholl	90^*^, 130^**^; 100, 120 μm	120 μm^*^	ns
	Nodes	Total	ns	**	^*^
		Sholl	ns	ns	ns
	Endings	Total	ns	^*^	**
		Sholl	ns	ns	90 μm^*^
	Av. diameter	Total	ns	ns	ns
		Sholl	ns	10 μm^***^	10 μm^***^
	Length	Total	ns	ns	ns
		Sholl	ns	120 μm^*^	ns
	Surface area	Total	ns	ns	ns
		Sholl	ns	120 μm^*^	ns
	Volume	Total	ns	ns	ns
		Sholl	ns	ns	ns
Apical arbor Segment analysis	Tortuosity	Branch	ns	O2^*^, O3, O5, O7	ns
		Terminal	O8^*^, O10	O8^*^, O10	ns
	Av. diameter	Branch	ns	ns	ns
		Terminal	ns	ns	ns
	Length	Branch	ns	O3^*^	O3^*^
		Terminal	ns	O6^*^	ns
	Surface area	Branch	ns	O3^*^	O3^*^
		Terminal	ns	ns	O7^*^
	Volume	Branch	ns	O3^*^	ns
		Terminal	O7^*^	ns	O7^*^

^*^ *P* < 0.05; ^**^P < 0.001; ^***^P < 0.0001.

**Table 2:** Summary of the analysis regarding differences observed during hibernation of the Syrian hamster concerning basal dendrites

			C vs. T(AT8−)	C vs. T(AT8+)	T(AT8−) vs. (AT8+)
Basal arbor Whole-cell reconstructions	Intersections	Total	ns	ns	ns
		Sholl	ns	ns	ns
	Nodes	Total	ns	ns	ns
		Sholl	ns	20^***^, 40 μm	20 μm^***^; 40 μm^*^
	Endings	Total	ns	ns	ns
		Sholl	ns	ns	ns
	Av. diameter	Total	ns	**	***
		Sholl	ns	20–30 μm^***^; 50–60 μm^**^; 40 μm^*^	20–60 μm^***^; 70 μm^*^
	Length	Total	ns	ns	ns
		Sholl	ns	ns	ns
	Surface area	Total	ns	ns	ns
		Sholl	ns	90 μm^*^	ns
	Volume	Total	ns	*	ns
		Sholl	ns	50–70 μm^**^; 80–100 μm^*^	50, 70 μm^**^; 60 μm^*^
Basal arbor Segment analysis	Tortuosity	Branch	O1^*^	ns	ns
		Terminal	O2^*^	ns	ns
	Av. diameter	Branch	ns	O1^**^; O2^*^	O1***
		Terminal	ns	ns	O2^*^; O3
	Length	Branch	ns	O1^***^; O3^*^	ns
		Terminal	ns	O2^***^; O3	O2^**^; O3^***^; O4^*^
	Surface area	Branch	ns	O1^***^	O1^***^; O2^*^; O3
		Terminal	ns	O2^***^; O3	O2^**^; O3^***^; O4^*^
	Volume	Branch	ns	O1^***^; O2^*^	O1^***^; O2^**^; O3^*^
		Terminal	ns	O2^***^; O3	O2^***^; O3

^*^ *P* < 0.05; ^**^P < 0.001; ^***^P < 0.0001.

**Table 3:** Summary of the analysis regarding differences observed during hibernation of the Syrian hamster concerning dendritic spine morphology of the apical main dendrite and basal dendrites

			C vs. T(AT8−)	C vs. T(AT8+)	T(AT8−) vs. (AT8+)
Apical arbor Whole-cell reconstructions	Density	Total	ns	ns	ns
		Sholl	100 μm*	ns	ns
	Length	Total	***	**	ns
		Sholl	60 μm^***^; 30, 40, 70, 130 μm^**^; 80 μm^*^	60 μm^*^	ns
	Area	Total	**	**	ns
		Sholl	ns	ns	ns
	Volume	Total	ns	**	ns
		Sholl	ns	150 μm^*^	ns
Apical arbor Segment analysis	Density	Total	ns	ns	ns
		Sholl	ns	ns	ns
	Length	Total	***	ns	ns
		Sholl	30 μm^***^; 50 μm^*^	ns	20, 30 μm^**^
	Area	Total	***	*	**
		Sholl	30, 40 μm^***^; 20, 50, 60, 130 μm^**^; 80, 120 μm^*^	ns	30, 40 μm^*^
	Volume	Total	***	*	ns
		Sholl	40 μm^***^; 30, 60, 130 μm^**^; 20, 80, 90, 120 μm^*^	ns	40 μm^*^

^*^ *P* < 0.05; ^**^P < 0.001; ^***^P < 0.0001.

## Discussion

### AT8-Immunoreactivity Pattern in the Syrian Hamster Somatosensory Cortex

During the hibernation of the Syrian hamster, some specific neuronal subpopulations are more prone to express phosphorylated tau. Making use of this, we studied cortical cells from layer Va with abundant AT8 labeling (in torpid animals; T(AT8+)) and layer Va cells that do not express hyperphosphorylated tau in either control animals or the torpor group T(AT8−). Comparing between groups allows us to study the effect of tau phosphorylation on the morphology of cortical neurons. However, we should take into account that, in these T(AT8−) cells, tau could be phosphorylated in residues other than Serines 202 and 205. In fact, using western Blot, Stieler and colleagues (Stieler et al. 2011) showed an overall increase in phosphorylated tau during torpor using the following antibodies: AT100 (T212/S214/T217), AT180 (T231/S235), AT270 (T181), and PHF1 (S396/S404). Moreover, it has been described that, in AD, neurofibrillary tangles labeled with the AT8 antibody could not be recognized by the AT100 antibody and vice versa (Regalado-Reyes et al. 2019). Therefore, studying if tau phosphorylation takes place simultaneously, in a sequential manner or independently within pyramidal neurons during hibernation, may shed light on the mechanisms that promote neuronal plasticity.

The majority of the studies carried out to date have analyzed the plastic changes in CA3 neurons upon hibernation (Popov et al. 1992; Magariños et al. 2006; Bullmann et al. 2016). In this regard, strong reactivity for AT8 was found in most of the hippocampal CA3 pyramidal cells, reflecting a more homogenous pattern than in the neocortex. In our study, since we were able to compare between cells that overexpressed phosphorylated tau and cells which did not, it was possible to evaluate whether the results obtained were specifically attributed to phosphorylation of the tau protein.

Here, we observed an increase in the basal dendrite diameter in AT8-positive cells. Changes in the diameter of the basal dendrites may affect calcium dynamics, since it has been reported that the peak calcium levels are inversely related to branch diameter (Anwar et al. 2014). This is important because calcium influx into the cytoplasm of dendrites and dendritic spines is involved in cytoskeletal remodeling, regulating dendrite morphogenesis (Higley and Sabatini 2008; Rosenberg and Spitzer 2011). Moreover, it has been suggested that hibernation promotes a decrease in the activity of calcium channels to prevent excessive Ca^2+^ entry (Gattoni and Bernocchi 2019; Wang et al. 2002; Zhang et al. 2020). Based on these data, we propose a relationship between tau hyperphosphorylation and calcium signaling by dendrite remodeling to prevent neurodegeneration. Future studies should focus on interpreting these findings from a functional perspective.

### The Protein Tau and Neuronal Plasticity of Neocortical Cells during Hibernation

The main finding of the present study is that tau phosphorylation may play a role in the morphological changes that take place in cortical pyramidal neurons during the hibernation of the Syrian hamster. We have shown that layer Va cells with abundant AT8 expression have fewer nodes, intersections, and endings (less complexity) in the apical dendrite. This is in agreement with other studies in CA3 neurons that describe a reduced apical dendritic tree complexity (Popov et al. 1992; Magariños et al. 2006; Bullmann et al. 2016). Different branch structures undertake distinct forms of neuronal processing within the dendritic tree before input potentials arrive at the soma. Therefore, there may be greater potential for compartmentalization in the cells that have a more highly branched pattern than in those with fewer branches (Stuart et al. 1997; Koch and Segev 2000; Spruston 2008).

The impact on cell function of the accumulation of phosphorylated tau protein is not clear. However, it has been suggested that high tortuosity of tau-positive neuronal cells may be related to excessive phosphorylation in the brain of dogs (Wegiel et al. 1998). This result is in line with previous studies that described a dendritic retraction in hibernating golden-mantled ground squirrels (von der Ohe et al. 2006) and neurite retraction in rat cerebellar granule neurons upon GSK-3-mediated hyperphosphorylation of the protein tau (Sayas et al. 2002). Our results are in agreement with the above data, as we observed that tortuosity was higher in the apical tree of T(AT8+) cells than in control cells.

Regarding the study of dendritic spines, we observed no spine density variation in the apical or basal dendrites of somatosensory layer Va neurons as a consequence of the hibernation of Syrian hamsters. Given that previous studies—using different methodological approaches—describe a lower spine number in CA1 and CA3 hippocampal neurons (Popov et al. 1992; Magariños et al. 2006; Bullmann et al. 2016), our results may suggest a brain region-dependent response upon hibernation between the hippocampal and somatosensory pyramidal cells.

Nevertheless, we observed that spines were significantly longer and larger during hibernation. This is in contrast to previous studies that show spine length reduction in hippocampal pyramidal neurons (Popov et al. 1992; Popov and Bocharova 1992; Magariños et al. 2006). Again, hippocampus and neocortex may respond differently when hibernation is triggered. Since the length of the dendritic spine is proportional to the extent of biochemical and electrical isolation from its parent dendrite and the spine size is correlated with synaptic currents (Harris and Stevens 1989; Yuste and Denk 1995; Nusser et al. 1998; Matsuzaki et al. 2004), the findings described here suggest a variation in the integration of inputs that may be regarded as a compensatory mechanism.

**Figure 18:**
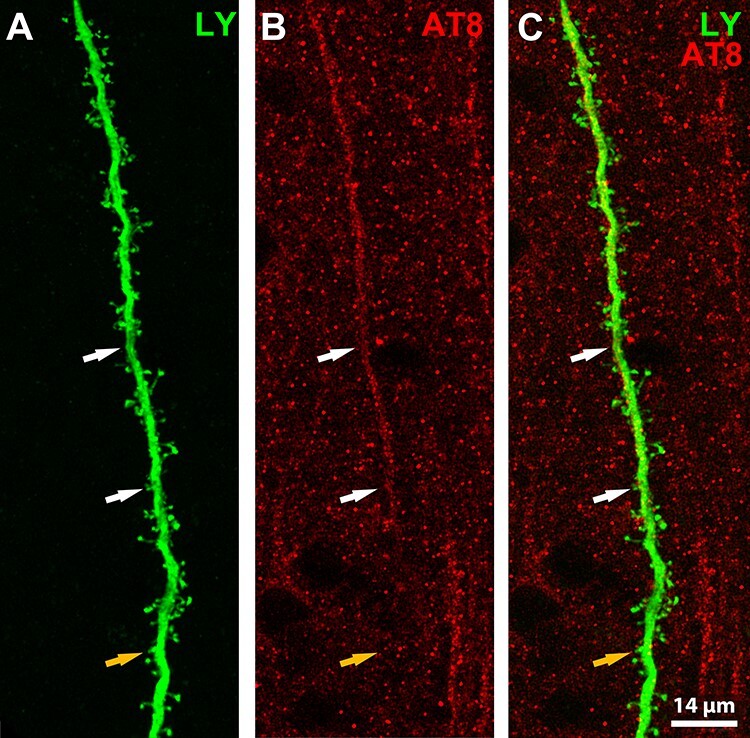
(*A*–*C*) Representative confocal image showing AT8 immunostaining in the distal region of the apical dendrite from an injected layer Va pyramidal cell. LY is shown in green and AT8 is shown in red. Orange arrows point at the dendrite segment where hyperphosphorylated tau is not detected and white arrows indicate regions of the dendrite where hyperphosphorylated tau is highly expressed. Scale bar, 14 μm.

Tau mislocalization may provide a valuable clue to explain and understand the role of Tau in synaptic alterations. Under pathological situations such as AD, misfolded tau has been detected at presynaptic and postsynaptic terminals, which may represent early signs of neuronal impairment (Tai et al. 2014). Later studies found hyperphosphorylated tau in the thorny excrescences of CA3 hippocampal neurons of AD patients (Blazquez-Llorca et al. 2011) and in the dendritic spines of CA3 neurons of the transgenic mouse model P301S (Hoffmann et al. 2013). Phosphorylated tau localization during the hibernation of Syrian hamsters is mainly restricted to the apical dendrite and the cellular soma. We have not detected phospho-tau in the dendritic spines (in somatosensory cortex or in the hippocampus). However, we have detected AT8 immunostaining in the distal region of the apical dendrite from injected layer Va pyramidal cells that do not contain phosphorylated tau in the soma or any other regions of the proximal apical or basal dendrites (Fig. 18). Future studies including the terminal tuft dendrites of pyramidal neurons should be performed to better understand the effects of tau hyperphosphorylation on layer V pyramidal neurons.

The large number of apical dendritic shafts present in layers II–III, which are labeled with AT8, contrasts with the low number of cells with such labeling in layers V–VI (see Fig. 2*B*). The most likely interpretation that explains this fact is that neurons that have hyperphosphorylated tau in distal regions of the apical dendrite do not present it in the soma, as shown in Figure 18.

Similar immunostaining was described previously in AD patients, with PHF-tauAT8 being found in the distal dendritic segments of Lucifer yellow-injected cells, classified as pattern IIa neurons (Merino-Serrais et al. 2013). Merino and colleagues suggested that dendritic spine loss is associated with the intracellular tau pathology, occurring first in the distal and then in the more proximal regions (Merino-Serrais et al. 2013). They also suggested that disconnection occurs only in the distal segments of pyramidal cells, where accumulation of fibrillary phospho-tauAT8 and reduction in the density of dendritic spines is observed. Further evidence of distal phospho-tau localization was described in a recent study carried out in AD samples, where AT8-ir segments were localized in the distal basal dendrites of isolated cortical neurons. This localization appears to follow a sequential pattern that could support the transcellular spreading of pathological “tau seeds,” a prion model of tau propagation (Braak and Del Tredici 2018; Perez et al. 2019). Our results show that this special phospho-tau localization is present under nonpathological conditions.

### Neuronal Activity and Morphological Cell Alterations during Syrian Hamster Hibernation

Torpor is characterized by a notable decrease in neuronal activity throughout the whole brain (reviewed in Sonntag and Arendt 2019). EEG studies at the end of the nineteenth century demonstrated that the cortex and other brain regions appear to remain silenced at low temperatures (Krelstein et al. 1990). This reduction, in parallel with the temperature drop, follows a progressive and sequential EEG frequency decrease, where the neocortex is affected first and the hippocampus last (Heller 1979; Curry-Hyde et al. 2020). However, the limbic system remains responsive to stimuli during hibernation, as CA1 hippocampal neurons are able to generate action potentials below 15 °C, even though they cannot generate long-term potentiation (LTP) (Hamilton et al. 2017; Horowitz and Horwitz 2019). It could be speculated that the differences in neuronal activity for each brain region might account for the differential neuroplasticity found between the hippocampus and somatosensory cortex neurons. Temperature drop during the course of hibernation is likely to be behind the general reduction of EEG signal (Cerri 2017); however, this point remains controversial. A recent study highlighted the maintenance of electrical properties of peripheral somatosensory neurons (dorsal root ganglia) during the hibernation of 13-lined ground squirrels (Hoffstaetter et al. 2018). Hoffstaetter suggested complex compensation mechanisms that involve a decrease in voltage-gated sodium channel activity and that cannot simply be explained by a decrease in temperature.

In situ hybridization studies documented that during hibernation, c-Fos expression is suppressed in the cortex of the 13-lined ground squirrel (Bratincsak et al. 2007). In addition, ^14^C-2-deoxyglucose uptake is also reduced in Golden-mantled ground squirrels (Kilduff et al. 1990). Both articles highlighted the activity inhibition of cortical regions in torpor, in line with the abovementioned electrophysiological studies. However, these 2 studies also showed activation of hypothalamic regions such as the suprachiasmatic nucleus, which may be responsible for promoting initiation of the arousal state. Another feature that could give us clues about cell activity is the organization of the Golgi apparatus, involved in the processing and transport of proteins. In this regard, torpor promotes a general reduction in the volume and surface area of the elements of Golgi apparatus in both the hippocampus and neocortex (Anton-Fernandez et al. 2015).

Despite the cortical neural inactivity in some brain regions, hibernating animals perceive temperature variations and external sensory stimuli, including touch and acoustic detection (Strumwasser 1959). This indicates that the sensory system is not fully silenced and remains interconnected to other brain areas.

Collectively, the molecular and electrophysiological studies clearly indicate that brain activity during hibernation is region-dependent. This could be related to the differential neuronal morphology described here and in previous work. Future in vivo studies are needed to examine the functionality of neuronal connectivity between brain regions during hibernation.

## Funding

Spanish “Ministerio de Ciencia, Innovación y Universidades” (grant PGC2018-094307-B-I00); Cajal Blue Brain Project (the Spanish partner of the Blue Brain Project initiative from EPFL, Switzerland); Centro de Investigación en Red sobre Enfermedades Neurodegenerativas (CIBERNED, CB06/05/0066, Spain).

## Notes

We would like to thank Lorena Valdes and Miriam Marin for technical assistance and Nick Guthrie for his helpful comments and editorial assistance. *Conflict of Interest*: None declared.

## Supplementary Material

Supplementary_material_tgaa018

## References

[ref1] Antón-Fernández A , Leon-Espinosa G, DeFelipe J, Munoz A. 2015. Changes in the Golgi apparatus of neocortical and hippocampal neurons in the hibernating hamster. Front Neuroanat. 9:157. 26696838 10.3389/fnana.2015.00157 PMC4678224

[ref2] Anwar H , Roome CJ, Nedelescu H, Chen W, Kuhn B, De Schutter E. 2014. Dendritic diameters affect the spatial variability of intracellular calcium dynamics in computer models. Front Cell Neurosci. 8:168. 25100945 10.3389/fncel.2014.00168 PMC4107854

[ref3] Arendt T , Stieler J, Strijkstra AM, Hut RA, Rudiger J, Van der Zee EA, Harkany T, Holzer M, Hartig W. 2003. Reversible paired helical filament-like phosphorylation of tau is an adaptive process associated with neuronal plasticity in hibernating animals. J Neurosci. 23:6972–6981. 12904458 10.1523/JNEUROSCI.23-18-06972.2003 PMC6740664

[ref4] Benavides-Piccione R , DeFelipe J. 2003. Different populations of tyrosine-hydroxylase-immunoreactive neurons defined by differential expression of nitric oxide synthase in the human temporal cortex. Cereb Cortex. 13:297–307. 12571119 10.1093/cercor/13.3.297

[ref5] Benavides-Piccione R , Hamzei-Sichani F, Ballesteros-Yanez I, DeFelipe J, Yuste R. 2006. Dendritic size of pyramidal neurons differs among mouse cortical regions. Cereb Cortex. 16:990–1001. 16195469 10.1093/cercor/bhj041

[ref5a] Benavides-Piccione R, Fernaud-Espinosa I, Robles V, Yuste R, DeFelipe J. 2013. Age-based comparison of human dendritic spine structure using complete three-dimensional reconstructions. Cereb Cortex. 23(8):1798–810. 22710613 10.1093/cercor/bhs154 PMC3698364

[ref5b] Benavides-Piccione R, Regalado-Reyes M, Fernaud-Espinosa I, Kastanauskaite A, Tapia-González S, León-Espinosa G, Rojo C, Insausti R, Segev I, DeFelipe J. 2020. Differential structure of hippocampal CA1 pyramidal neurons in the human and mouse. Cereb Cortex. 30(2):730–752. 31268532 10.1093/cercor/bhz122

[ref6] Blazquez-Llorca L , Garcia-Marin V, Merino-Serrais P, Avila J, DeFelipe J. 2011. Abnormal tau phosphorylation in the thorny excrescences of CA3 hippocampal neurons in patients with Alzheimer's disease. J Alzheimers Dis. 26:683–698. 21677375 10.3233/JAD-2011-110659

[ref7] Braak H , Braak E. 1995. Staging of Alzheimer's disease-related neurofibrillary changes. Neurobiol Aging. 16:271–278 discussion 278–284. 7566337 10.1016/0197-4580(95)00021-6

[ref8] Braak H , Del Tredici K. 2018. Spreading of tau pathology in sporadic Alzheimer's disease along Cortico-cortical top-down connections. Cereb Cortex. 28:3372–3384. 29982389 10.1093/cercor/bhy152 PMC6095209

[ref9] Bratincsak A , McMullen D, Miyake S, Toth ZE, Hallenbeck JM, Palkovits M. 2007. Spatial and temporal activation of brain regions in hibernation: c-fos expression during the hibernation bout in thirteen-lined ground squirrel. J Comp Neurol. 505:443–458. 17912746 10.1002/cne.21507 PMC2774134

[ref10] Bullmann T , Seeger G, Stieler J, Hanics J, Reimann K, Kretzschmann TP, Hilbrich I, Holzer M, Alpar A, Arendt T. 2016. Tau phosphorylation-associated spine regression does not impair hippocampal-dependent memory in hibernating golden hamsters. Hippocampus. 26:301–318. 26332578 10.1002/hipo.22522

[ref11] Cerri M . 2017. Consciousness in hibernation and synthetic torpor. J Integr Neurosci. 16:S19–S26. 29125496 10.3233/JIN-170063

[ref12] Chayama Y , Ando L, Tamura Y, Miura M, Yamaguchi Y. 2016. Decreases in body temperature and body mass constitute pre-hibernation remodelling in the Syrian golden hamster, a facultative mammalian hibernator. R Soc Open Sci. 3:160002. 27152216 10.1098/rsos.160002 PMC4852639

[ref13] Curry-Hyde A , Ueberham U, Arendt T, Janitz M. 2020. Neural circular transcriptomes across mammalian species. Genomics. 112(2):1162–1166. 31255695 10.1016/j.ygeno.2019.06.030

[ref14] DeFelipe J , Fariñas I. 1992. The pyramidal neuron of the cerebral cortex: morphological and chemical characteristics of the synaptic inputs. Prog Neurobiol. 39:563–607. 1410442 10.1016/0301-0082(92)90015-7

[ref15] Elston GN , Benavides-Piccione R, DeFelipe J. 2001. The pyramidal cell in cognition: a comparative study in human and monkey. J Neurosci. 21:RC163. 11511694 10.1523/JNEUROSCI.21-17-j0002.2001 PMC6763111

[ref16] Gattoni G , Bernocchi G. 2019. Calcium-binding proteins in the nervous system during hibernation: neuroprotective strategies in hypometabolic conditions? Int J Mol Sci. 20:2364. 10.3390/ijms20092364 PMC6540041 31086053

[ref17] Gong CX , Grundke-Iqbal I, Iqbal K. 2010. Targeting tau protein in Alzheimer's disease. Drugs Aging. 27:351–365. 20450234 10.2165/11536110-000000000-00000

[ref18] Hamilton JS , Chau SM, Malins KJ, Ibanez GG, Horowitz JM, Horwitz BA. 2017. Syrian hamster neuroplasticity mechanisms fail as temperature declines to 15 °C, but histaminergic neuromodulation persists. J Comp Physiol B. 187:779–791. 28391591 10.1007/s00360-017-1078-5

[ref19] Harris KM , Stevens JK. 1989. Dendritic spines of CA 1 pyramidal cells in the rat hippocampus: serial electron microscopy with reference to their biophysical characteristics. J Neurosci. 9:2982–2997. 2769375 10.1523/JNEUROSCI.09-08-02982.1989 PMC6569708

[ref20] Heller HC . 1979. Hibernation: neural aspects. Annu Rev Physiol. 41:305–321. 373593 10.1146/annurev.ph.41.030179.001513

[ref21] Higley MJ , Sabatini BL. 2008. Calcium signaling in dendrites and spines: practical and functional considerations. Neuron. 59:902–913. 18817730 10.1016/j.neuron.2008.08.020

[ref22] Hoffmann NA , Dorostkar MM, Blumenstock S, Goedert M, Herms J. 2013. Impaired plasticity of cortical dendritic spines in P301S tau transgenic mice. Acta Neuropathol Commun. 1:82. 24344647 10.1186/2051-5960-1-82 PMC3880070

[ref23] Hoffstaetter LJ , Mastrotto M, Merriman DK, Dib-Hajj SD, Waxman SG, Bagriantsev SN, Gracheva EO. 2018. Somatosensory neurons enter a state of altered excitability during hibernation. Curr Biol. 28(2998–3004):e2993. 10.1016/j.cub.2018.07.020 PMC6173314 30174191

[ref24] Hoover BR , Reed MN, Su J, Penrod RD, Kotilinek LA, Grant MK, Pitstick R, Carlson GA, Lanier LM, Yuan LL, et al. 2010. Tau mislocalization to dendritic spines mediates synaptic dysfunction independently of neurodegeneration. Neuron. 68:1067–1081. 21172610 10.1016/j.neuron.2010.11.030 PMC3026458

[ref25] Horowitz JM , Horwitz BA. 2019. Extreme neuroplasticity of hippocampal CA1 pyramidal neurons in hibernating mammalian species. Front Neuroanat. 13:9. 30814935 10.3389/fnana.2019.00009 PMC6381046

[ref26] Iqbal K , Liu F, Gong CX. 2016. Tau and neurodegenerative disease: the story so far. Nat Rev Neurol. 12:15–27. 26635213 10.1038/nrneurol.2015.225

[ref27] Ittner A , Ittner LM. 2018. Dendritic tau in Alzheimer's disease. Neuron. 99:13–27. 30001506 10.1016/j.neuron.2018.06.003

[ref28] Kanari L , Ramaswamy S, Shi Y, Morand S, Meystre J, Perin R, Abdellah M, Wang Y, Hess K, Markram H. 2019. Objective morphological classification of neocortical pyramidal cells. Cereb Cortex. 29:1719–1735. 30715238 10.1093/cercor/bhy339 PMC6418396

[ref29] Kilduff TS , Miller JD, Radeke CM, Sharp FR, Heller HC. 1990. 14C-2-deoxyglucose uptake in the ground squirrel brain during entrance to and arousal from hibernation. J Neurosci. 10:2463–2475. 2376782 10.1523/JNEUROSCI.10-07-02463.1990 PMC6570375

[ref30] Koch C , Poggio T, Torre V. 1982. Retinal ganglion cells: a functional interpretation of dendritic morphology. Philos Trans R Soc Lond Ser B Biol Sci. 298:227–263. 6127730 10.1098/rstb.1982.0084

[ref31] Koch C , Segev I. 2000. The role of single neurons in information processing. Nat Neurosci. 3(Suppl):1171–1177. 11127834 10.1038/81444

[ref32] Krelstein MS , Thomas MP, Horowitz JM. 1990. Thermal effects on long-term potentiation in the hamster hippocampus. Brain Res. 520:115–122. 2207625 10.1016/0006-8993(90)91696-e

[ref33] Leon-Espinosa G , Regalado-Reyes M, DeFelipe J, Muñoz A. 2017. Changes in neocortical and hippocampal microglial cells during hibernation. Brain Struct Funct. 223:1881–1895. 29260372 10.1007/s00429-017-1596-7

[ref34] Leon-Espinosa G , Anton-Fernandez A, Tapia-Gonzalez S, DeFelipe J, Muñoz A. 2018. Modifications of the axon initial segment during the hibernation of the Syrian hamster. Brain Struct Funct. 223:4307–4321. 30219944 10.1007/s00429-018-1753-7

[ref35] Leon-Espinosa G , DeFelipe J, Muñoz A. 2019. The Golgi apparatus of neocortical glial cells during hibernation in the Syrian hamster. Front Neuroanat. 13:92. 31824270 10.3389/fnana.2019.00092 PMC6882278

[ref36] Magariños AM , McEwen BS, Saboureau M, Pevet P. 2006. Rapid and reversible changes in intrahippocampal connectivity during the course of hibernation in European hamsters. Proc Natl Acad Sci U S A. 103:18775–18780. 17121986 10.1073/pnas.0608785103 PMC1693738

[ref37] Matsuzaki M , Honkura N, Ellis-Davies GC, Kasai H. 2004. Structural basis of long-term potentiation in single dendritic spines. Nature. 429:761–766. 15190253 10.1038/nature02617 PMC4158816

[ref38] Medina M , Hernandez F, Avila J. 2016. New features about tau function and dysfunction. Biomolecules. 6(2):21. 10.3390/biom6020021 PMC4919916 27104579

[ref39] Merino-Serrais P , Benavides-Piccione R, Blazquez-Llorca L, Kastanauskaite A, Rabano A, Avila J, DeFelipe J. 2013. The influence of phospho-tau on dendritic spines of cortical pyramidal neurons in patients with Alzheimer's disease. Brain. 136:1913–1928. 23715095 10.1093/brain/awt088 PMC3673457

[ref40] Morin LP , Wood RI. 2001. A Stereotaxic Atlas of the Golden Hamster Brain. San Diego (CA): Academic Press.

[ref41] Nelson PT , Alafuzoff I, Bigio EH, Bouras C, Braak H, Cairns NJ, Castellani RJ, Crain BJ, Davies P, Del Tredici K, et al. 2012. Correlation of Alzheimer disease neuropathologic changes with cognitive status: a review of the literature. J Neuropathol Exp Neurol. 71:362–381. 22487856 10.1097/NEN.0b013e31825018f7 PMC3560290

[ref42] Nusser Z , Lujan R, Laube G, Roberts JD, Molnar E, Somogyi P. 1998. Cell type and pathway dependence of synaptic AMPA receptor number and variability in the hippocampus. Neuron. 21:545–559. 9768841 10.1016/s0896-6273(00)80565-6

[ref43] Perez M , Avila J, Hernandez F. 2019. Propagation of tau via extracellular vesicles. Front Neurosci. 13:698. 31312118 10.3389/fnins.2019.00698 PMC6614378

[ref44] Popov VI , Bocharova LS. 1992. Hibernation-induced structural changes in synaptic contacts between mossy fibres and hippocampal pyramidal neurons. Neuroscience. 48:53–62. 1584425 10.1016/0306-4522(92)90337-2

[ref45] Popov VI , Bocharova LS, Bragin AG. 1992. Repeated changes of dendritic morphology in the hippocampus of ground squirrels in the course of hibernation. Neuroscience. 48:45–51. 1584424 10.1016/0306-4522(92)90336-z

[ref46] Popov VI , Medvedev NI, Patrushev IV, Ignat'ev DA, Morenkov ED, Stewart MG. 2007. Reversible reduction in dendritic spines in CA1 of rat and ground squirrel subjected to hypothermia-normothermia in vivo: a three-dimensional electron microscope study. Neuroscience. 149:549–560. 17919827 10.1016/j.neuroscience.2007.07.059

[ref46a] Regalado-Reyes M, Furcila D, Hernández F, Ávila J, DeFelipe J, León-Espinosa G. 2019. Phospho-Tau changes in the human CA1 during Alzheimer's disease progression. J Alzheimers Dis. 69(1):277–288. 30958368 10.3233/JAD-181263 PMC6598029

[ref47] Rosenberg SS , Spitzer NC. 2011. Calcium Signaling in neuronal development. Cold Spring Harb Perspect Biol. 3:10. 10.1101/cshperspect.a004259 PMC3179332 21730044

[ref48] Ruf T , Geiser F. 2015. Daily torpor and hibernation in birds and mammals. Biol Rev Camb Philos Soc. 90:891–926. 25123049 10.1111/brv.12137 PMC4351926

[ref48a] Sayas CL, Avila J, Wandosell F. 2002. Glycogen synthase kinase-3 is activated in neuronal cells by Ga12 and Ga13 by Rho-independent and Rho-dependent mechanisms. Journal of Neuroscience. 22(16):6863–6875. 12177184 10.1523/JNEUROSCI.22-16-06863.2002 PMC6757878

[ref49] Shepherd GM , Brayton RK, Miller JP, Segev I, Rinzel J, Rall W. 1985. Signal enhancement in distal cortical dendrites by means of interactions between active dendritic spines. Proc Natl Acad Sci U S A. 82:2192–2195. 3856892 10.1073/pnas.82.7.2192 PMC397519

[ref50] Sonntag M , Arendt T. 2019. Neuronal activity in the hibernating brain. Front Neuroanat. 13:71. 31338028 10.3389/fnana.2019.00071 PMC6629779

[ref51] Sotiropoulos I , Galas MC, Silva JM, Skoulakis E, Wegmann S, Maina MB, Blum D, Sayas CL, Mandelkow EM, Mandelkow E, et al. 2017. Atypical, non-standard functions of the microtubule associated tau protein. Acta Neuropathol Commun. 5:91. 29187252 10.1186/s40478-017-0489-6 PMC5707803

[ref52] Spruston N. 2008. Pyramidal neurons: dendritic structure and synaptic integration. Nat Rev Neurosci. 9:206–221. 18270515 10.1038/nrn2286

[ref53] Stieler JT , Bullmann T, Kohl F, Toien O, Bruckner MK, Hartig W, Barnes BM, Arendt T. 2011. The physiological link between metabolic rate depression and tau phosphorylation in mammalian hibernation. PLoS One. 6:e14530. 21267079 10.1371/journal.pone.0014530 PMC3022585

[ref54] Strumwasser F . 1959. Thermoregulatory, brain and behavioral mechanisms during entrance into hibernation in the squirrel. Citellus beecheyi. Am J Physiol. 196:15–22. 13617428 10.1152/ajplegacy.1958.196.1.15

[ref55] Stuart G , Spruston N, Sakmann B, Hausser M. 1997. Action potential initiation and backpropagation in neurons of the mammalian CNS. Trends Neurosci. 20:125–131. 9061867 10.1016/s0166-2236(96)10075-8

[ref56] Tai HC , Wang BY, Serrano-Pozo A, Frosch MP, Spires-Jones TL, Hyman BT. 2014. Frequent and symmetric deposition of misfolded tau oligomers within presynaptic and postsynaptic terminals in Alzheimer's disease. Acta Neuropathol Commun. 2:146. 25330988 10.1186/s40478-014-0146-2 PMC4209049

[ref57] von der Ohe CG , Darian-Smith C, Garner CC, Heller HC. 2006. Ubiquitous and temperature-dependent neural plasticity in hibernators. J Neurosci. 26:10590–10598. 17035545 10.1523/JNEUROSCI.2874-06.2006 PMC6674705

[ref58] von der Ohe CG , Garner CC, Darian-Smith C, Heller HC. 2007. Synaptic protein dynamics in hibernation. J Neurosci. 27:84–92. 17202475 10.1523/JNEUROSCI.4385-06.2007 PMC6672296

[ref59] Wang Y , Mandelkow E. 2016. Tau in physiology and pathology. Nat Rev Neurosci. 17:5–21. 26631930 10.1038/nrn.2015.1

[ref60] Wang SQ , Lakatta EG, Cheng H, Zhou ZQ. 2002. Adaptive mechanisms of intracellular calcium homeostasis in mammalian hibernators. J Exp Biol. 205:2957–2962. 12200399 10.1242/jeb.205.19.2957

[ref61] Weingarten MD , Lockwood AH, Hwo SY, Kirschner MW. 1975. A protein factor essential for microtubule assembly. Proc Natl Acad Sci U S A. 72:1858–1862. 1057175 10.1073/pnas.72.5.1858 PMC432646

[ref61a] Wegiel J, Wisniewski HM, Soltysiak. 1998. Region- and cell-type-specific pattern of tau phosphorylation in dog brain. Brain Res. 802(1–2):259–66. 9748620 10.1016/s0006-8993(98)00542-3

[ref62] Yoshiyama Y , Higuchi M, Zhang B, Huang SM, Iwata N, Saido TC, Maeda J, Suhara T, Trojanowski JQ, Lee VM. 2007. Synapse loss and microglial activation precede tangles in a P301S tauopathy mouse model. Neuron. 53:337–351. 17270732 10.1016/j.neuron.2007.01.010

[ref63] Yuste R , Denk W. 1995. Dendritic spines as basic functional units of neuronal integration. Nature. 375:682–684. 7791901 10.1038/375682a0

[ref64] Zhang J , Li X, Ismail F, Xu S, Wang Z, Peng X, Yang C, Chang H, Wang H, Gao Y. 2020. Priority strategy of intracellular Ca^2+^ homeostasis in skeletal muscle fibers during the multiple stresses of hibernation. Cell. 9:42. 10.3390/cells9010042 PMC7016685 31877883

