# Supplementary material for: Effect of Phosphorylated Tau on Cortical Pyramidal Neuron Morphology during Hibernation
Source: Cereb Cortex Commun. 2020 May 21;1(1):tgaa018. doi: 10.1093/texcom/tgaa018 (PMC8152943; doi:10.1093/texcom/tgaa018)
Supplement: Supplementary_material_tgaa018 [file supplementary_material_tgaa018.docx]

| **Supplementary table 1\| Apical arbor intersectionsSholl analysis** | | | |  | **Supplementary table 2\| Apical arbor intersections mean total values** | | | |
| --- | --- | --- | --- | --- | --- | --- | --- | --- |
| Distance from soma (µm) | C vs T(AT8-) | C vs T(AT8+) | T(AT8-) vs T(AT8+) |  | K=11.60 | C | T(AT8-) | T(AT8+) |
| 0 | ns | ns | ns |  | C |  | ** | * |
| 10 | ns | ns | ns |  | T(AT8-) |  |  | ns |
| 20 | ns | ns | ns |  | T(AT8+) |  |  |  |
| 30 | ns | ns | ns |  |  |  |  |  |
| 40 | ns | ns | ns |  |  |  |  |  |
| 50 | ns | ns | ns |  |  |  |  |  |
| 60 | ns | ns | ns |  |  |  |  |  |
| 70 | ns | ns | ns |  |  |  |  |  |
| 80 | ns | ns | ns |  |  |  |  |  |
| 90 | * | ns | ns |  | **Supplementary table 3\| Apical arbor nodes mean total values** | | | |
| 100 | ** | ns | ns |  | F=6.507 | C | T(AT8-) | T(AT8+) |
| 110 | ns | ns | ns |  | C |  | ns | ** |
| 120 | ** | * | ns |  | T(AT8-) |  |  | * |
| 130 | * | ns | ns |  | T(AT8+) |  |  |  |
| 140 | ns | ns | ns |  |  |  |  |  |
| 150 | ns | ns | ns |  |  |  |  |  |
| 160 | ns | ns | ns |  |  |  |  |  |
| 170 | ns | ns | ns |  |  |  |  |  |
| 180 | ns | ns | ns |  |  |  |  |  |
|  |  |  |  |  |  |  |  |  |

| **Supplementary table 4\| Apical arbor endingsSholl analysis** | | | |  | **Supplementary table 5\| Apical arbor endings mean total values** | | | |
| --- | --- | --- | --- | --- | --- | --- | --- | --- |
| Distance from soma (µm) | C vs T(AT8-) | C vs T(AT8+) | T(AT8-) vs T(AT8+) |  | K=8.635 | C | T(AT8-) | T(AT8+) |
| 0 | ns | ns | ns |  | C |  | ns | * |
| 10 | ns | ns | ns |  | T(AT8-) |  |  | ** |
| 20 | ns | ns | ns |  | T(AT8+) |  |  |  |
| 30 | ns | ns | ns |  |  |  |  |  |
| 40 | ns | ns | ns |  |  |  |  |  |
| 50 | ns | ns | ns |  |  |  |  |  |
| 60 | ns | ns | ns |  |  |  |  |  |
| 70 | ns | ns | ns |  |  |  |  |  |
| 80 | ns | ns | ns |  |  |  |  |  |
| 90 | ns | ns | * |  |  |  |  |  |
| 100 | ns | ns | ns |  |  |  |  |  |
| 110 | ns | ns | ns |  |  |  |  |  |
| 120 | ns | ns | ns |  |  |  |  |  |
| 130 | ns | ns | ns |  |  |  |  |  |
| 140 | ns | ns | ns |  |  |  |  |  |
| 150 | ns | ns | ns |  |  |  |  |  |
| 160 | ns | ns | ns |  |  |  |  |  |
| 170 | ns | ns | ns |  |  |  |  |  |
| 180 | ns | ns | ns |  |  |  |  |  |
|  |  |  |  |  |  |  |  |  |
| **Supplementary table 6\| Apical arbor average diameter Sholl analysis** | | | |  |  |  |  |  |
| Distance from soma (µm) | C vs T(AT8-) | C vs T(AT8+) | T(AT8-) vs T(AT8+) |  |  |  |  |  |
| 0 | ns | ns | ns |  |  |  |  |  |
| 10 | ns | *** | *** |  |  |  |  |  |
| 20 | ns | ns | ns |  |  |  |  |  |
| 30 | ns | ns | ns |  |  |  |  |  |
| 40 | ns | ns | ns |  |  |  |  |  |
| 50 | ns | ns | ns |  |  |  |  |  |
| 60 | ns | ns | ns |  |  |  |  |  |
| 70 | ns | ns | ns |  |  |  |  |  |
| 80 | ns | ns | ns |  |  |  |  |  |
| 90 | ns | ns | ns |  |  |  |  |  |
| 100 | ns | ns | ns |  |  |  |  |  |
| 110 | ns | ns | ns |  |  |  |  |  |
| 120 | ns | ns | ns |  |  |  |  |  |
| 130 | ns | ns | ns |  |  |  |  |  |
| 140 | ns | ns | ns |  |  |  |  |  |
| 150 | ns | ns | ns |  |  |  |  |  |
| 160 | ns | ns | ns |  |  |  |  |  |
| 170 | ns | ns | ns |  |  |  |  |  |
| 180 | ns | ns | ns |  |  |  |  |  |

| **Supplementary table 7\| Apical arbor length Sholl analysis** | | | | |
| --- | --- | --- | --- | --- |
| Distance from soma (µm) | | C vs T(AT8-) | C vs T(AT8+) | T(AT8-) vs T(AT8+) |
| 0 | | ns | ns | ns |
| 10 | | ns | ns | ns |
| 20 | | ns | ns | ns |
| 30 | | ns | ns | ns |
| 40 | | ns | ns | ns |
| 50 | | ns | ns | ns |
| 60 | | ns | ns | ns |
| 70 | | ns | ns | ns |
| 80 | | ns | ns | ns |
| 90 | | ns | ns | ns |
| 100 | | ns | ns | ns |
| 110 | | ns | ns | ns |
| 120 | | ns | * | ns |
| 130 | | ns | ns | ns |
| 140 | | ns | ns | ns |
| 150 | | ns | ns | ns |
| 160 | | ns | ns | ns |
| 170 | | ns | ns | ns |
| 180 | | ns | ns | ns |
|  | |  |  |  |
| **Supplementary table 8\| Apical arbor average surface areaSholl analysis** | | | | |
| Distance from soma (µm) | | C vs T(AT8-) | C vs T(AT8+) | T(AT8-) vs T(AT8+) |
| 0 | | ns | ns | ns |
| 10 | | ns | ns | ns |
| 20 | | ns | ns | ns |
| 30 | | ns | ns | ns |
| 40 | | ns | ns | ns |
| 50 | | ns | ns | ns |
| 60 | | ns | ns | ns |
| 70 | | ns | ns | ns |
| 80 | | ns | ns | ns |
| 90 | | ns | ns | ns |
| 100 | | ns | ns | ns |
| 110 | | ns | ns | ns |
| 120 | | ns | * | ns |
| 130 | | ns | ns | ns |
| 140 | | ns | ns | ns |
| 150 | | ns | ns | ns |
| 160 | | ns | ns | ns |
| 170 | | ns | ns | ns |
| 180 | | ns | ns | ns |
|  |  |  |  |  |
|  |  |  |  |  |

| **Supplementary table 9\| Basal arbor nodesSholl analysis** | | | |  |  |  |  |  |
| --- | --- | --- | --- | --- | --- | --- | --- | --- |
| Distance from soma (µm) | C vs T(AT8-) | C vs T(AT8+) | T(AT8-) vs T(AT8+) |  |  |  |  |  |
| 10 |  |  |  |  |  |  |  |  |
| 20 | ns | *** | *** |  |  |  |  |  |
| 30 | ns | ns | ns |  |  |  |  |  |
| 40 | ns | *** | * |  |  |  |  |  |
| 50 | ns | ns | ns |  |  |  |  |  |
| 60 | ns | ns | ns |  |  |  |  |  |
| 70 | ns | ns | ns |  |  |  |  |  |
| 80 | ns | ns | ns |  |  |  |  |  |
| 90 | ns | ns | ns |  |  |  |  |  |
| 100 | ns | ns | ns |  |  |  |  |  |
| 110 | ns | ns | ns |  |  |  |  |  |
| 120 | ns | ns | ns |  |  |  |  |  |
| 130 | ns | ns | ns |  |  |  |  |  |
| 140 | ns | ns | ns |  |  |  |  |  |
| 150 | ns | ns | ns |  |  |  |  |  |

| **Supplementary table 10\| Basal arbor average diameter Sholl analysis** | | | | | | | | |  | | **Supplementary table 11\| Basal arbor average diametermean total values** | | | | |
| --- | --- | --- | --- | --- | --- | --- | --- | --- | --- | --- | --- | --- | --- | --- | --- |
| Distance from soma (µm) | | C vs T(AT8-) | | C vs T(AT8+) | | T(AT8-) vs T(AT8+) | | |  | | K=14.73 | C | | T(AT8-) | T(AT8+) |
| 10 | |  | |  | |  | | |  | | C |  | | ns | ** |
| 20 | | ns | | *** | | *** | | |  | | T(AT8-) |  | |  | *** |
| 30 | | ns | | *** | | *** | | |  | | T(AT8+) |  | |  |  |
| 40 | | ns | | * | | *** | | |  | |  |  | |  |  |
| 50 | | ns | | ** | | *** | | |  | |  |  | |  |  |
| 60 | | ns | | ** | | *** | | |  | |  |  | |  |  |
| 70 | | ns | | ns | | * | | |  | |  |  | |  |  |
| 80 | | ns | | ns | | ns | | |  | |  |  | |  |  |
| 90 | | ns | | ns | | ns | | |  | |  |  | |  |  |
| 100 | | ns | | ns | | ns | | |  | |  |  | |  |  |
| 110 | | ns | | ns | | ns | | |  | |  |  | |  |  |
| 120 | | ns | | ns | | ns | | |  | |  |  | |  |  |
| 130 | | ns | | ns | | ns | | |  | |  |  | |  |  |
| 140 | | ns | | ns | | ns | | |  | |  |  | |  |  |
| 150 | | ns | | ns | | ns | | |  | |  |  | |  |  |
|  | |  | |  | | |  | |  |  | |  |  |  |  |
|  | |  | |  | |  | | |  | |  |  | |  |  |
| **Supplementary table 12\| Basal arbor surface areaSholl analysis** | | | | | | | | |  | |  |  |  |  |  |
| Distance from soma (µm) | | C vs T(AT8-) | | C vs T(AT8+) | | T(AT8-) vs T(AT8+) | | |  | |  |  |  |  |  |
| 10 | |  | |  | |  | | |  | |  |  |  |  |  |
| 20 | | ns | | ns | | ns | | |  | |  |  |  |  |  |
| 30 | | ns | | ns | | ns | | |  | |  |  |  |  |  |
| 40 | | ns | | ns | | ns | | |  | |  |  | |  |  |
| 50 | | ns | | ns | | ns | | |  | |  |  | |  |  |
| 60 | | ns | | ns | | ns | | |  | |  |  | |  |  |
| 70 | | ns | | ns | | ns | | |  | |  |  | |  |  |
| 80 | | ns | | ns | | ns | | |  | |  |  | |  |  |
| 90 | | ns | | * | | ns | | |  | |  |  | |  |  |
| 100 | | ns | | ns | | ns | | |  | |  |  | |  |  |
| 110 | | ns | | ns | | ns | | |  | |  |  | |  |  |
| 120 | | ns | | ns | | ns | | |  | |  |  | |  |  |
| 130 | | ns | | ns | | ns | | |  | |  |  | |  |  |
| 140 | | ns | | ns | | ns | | |  | |  |  | |  |  |
| 150 | | ns | | ns | | ns | | |  | |  |  | |  |  |
|  |  | |  | |  | |  |  | |  | | |  |  |  |
|  |  |  |  |  |  |  |  |  |  |  |  |  |  |  |  |
| **Supplementary table 13\| Basal arbor volume Sholl analysis** | | | | | | | | |  | | **Supplementary table 14\| Basal arbor volumemean total values** | | | | |
| Distance from soma (µm) | | C vs T(AT8-) | | C vs T(AT8+) | | T(AT8-) vs T(AT8+) | | |  | | 5.44 | C | | T(AT8-) | T(AT8+) |
| 10 | |  | |  | |  | | |  | | C |  | | ns | * |
| 20 | | ns | | ns | | ns | | |  | | T(AT8-) |  | |  | ns |
| 30 | | ns | | ns | | ns | | |  | | T(AT8+) |  | |  |  |
| 40 | | ns | | ns | | ns | | |  | |  |  | |  |  |
| 50 | | ns | | ** | | ** | | |  | |  |  | |  |  |
| 60 | | ns | | ** | | * | | |  | |  |  | |  |  |
| 70 | | ns | | ** | | ** | | |  | |  |  | |  |  |
| 80 | | ns | | * | | ns | | |  | |  |  | |  |  |
| 90 | | ns | | * | | ns | | |  | |  |  | |  |  |
| 100 | | ns | | * | | ns | | |  | |  |  | |  |  |
| 110 | | ns | | ns | | ns | | |  | |  |  | |  |  |
| 120 | | ns | | ns | | ns | | |  | |  |  | |  |  |
| 130 | | ns | | ns | | ns | | |  | |  |  | |  |  |
| 140 | | ns | | ns | | ns | | |  |  |  |  |  |  |  |
| 150 | | ns | | ns | | ns | | |  |  |  |  |  |  |  |

| **Supplementary table 15\|Apical arbor branching segments tortuosity** | | | |
| --- | --- | --- | --- |
| Order 2  K= 7,668 | C | T(AT8-) | T(AT8+) |
| C |  | ns | * |
| T(AT8-) |  |  | ns |
| T(AT8+) |  |  |  |
| Order 3  K= 6,689 | C | T(AT8-) | T(AT8+) |
| C |  | ns | * |
| T(AT8-) |  |  | ns |
| T(AT8+) |  |  |  |
| Order 5  K= 7,866 | C | T(AT8-) | T(AT8+) |
| C |  | ns | * |
| T(AT8-) |  |  | ns |
| T(AT8+) |  |  |  |
| Order 7  K= 7,763 | C | T(AT8-) | T(AT8+) |
| C |  | ns | * |
| T(AT8-) |  |  | ns |
| T(AT8+) |  |  |  |

| **Supplementary table 16\|Apical arbor branching segments length** | | | |
| --- | --- | --- | --- |
| Order 3  K= 9,887 | C | T(AT8-) | T(AT8+) |
| C |  | ns | * |
| T(AT8-) |  |  | * |
| T(AT8+) |  |  |  |

| **Supplementary table 17\|Apical arbor branching segments surface area** | | | |
| --- | --- | --- | --- |
| Order 3  K= 9,401 | C | T(AT8-) | T(AT8+) |
| C |  | ns | * |
| T(AT8-) |  |  | * |
| T(AT8+) |  |  |  |

| **Supplementary table 18\|\|Apical arbor branching segments volume** | | | |
| --- | --- | --- | --- |
| Order 3  K= 7,281 | C | T(AT8-) | T(AT8+) |
| C |  | ns | * |
| T(AT8-) |  |  | ns |
| T(AT8+) |  |  |  |

| **Supplementary table 19\|Apical arbor terminal segments tortuosity** | | | |
| --- | --- | --- | --- |
| Order 8  K= 11,49 | C | T(AT8-) | T(AT8+) |
| C |  | * | * |
| T(AT8-) |  |  | ns |
| T(AT8+) |  |  |  |
| Order 10  K= 10,15 | C | T(AT8-) | T(AT8+) |
| C |  | * | * |
| T(AT8-) |  |  | ns |
| T(AT8+) |  |  |  |

| **Supplementary table 20\|Apical arbor terminal segments length** | | | |
| --- | --- | --- | --- |
| Order 6  K= 5,816 | C | T(AT8-) | T(AT8+) |
| C |  | ns | * |
| T(AT8-) |  |  | ns |
| T(AT8+) |  |  |  |

| **Supplementary table 21\|Apical arbor terminal segments surface area** | | | |
| --- | --- | --- | --- |
| Order 7  K= 8,853 | C | T(AT8-) | T(AT8+) |
| C |  | ns | ns |
| T(AT8-) |  |  | * |
| T(AT8+) |  |  |  |

| **Supplementary table 22\|Apical arbor terminal segments volume** | | | |
| --- | --- | --- | --- |
| Order 7  K= 10,60 | C | T(AT8-) | T(AT8+) |
| C |  | * | ns |
| T(AT8-) |  |  | * |
| T(AT8+) |  |  |  |

| **Supplementary table 23\|Basal arbor branching segments tortuosity** | | | |
| --- | --- | --- | --- |
| Order 1  K= 6,628 | C | T(AT8-) | T(AT8+) |
| C |  | * | ns |
| T(AT8-) |  |  | ns |
| T(AT8+) |  |  |  |

| **Supplementary table 24\|Basal arbor branching segments diameter** | | | |
| --- | --- | --- | --- |
| Order 1  K= 16,87 | C | T(AT8-) | T(AT8+) |
| C |  | ns | ** |
| T(AT8-) |  |  | *** |
| T(AT8+) |  |  |  |
| Order 2  K= 15,63 | C | T(AT8-) | T(AT8+) |
| C |  | ns | * |
| T(AT8-) |  |  | ns |
| T(AT8+) |  |  |  |

| **Supplementary table 25\|Basal arbor branching segments length** | | | |
| --- | --- | --- | --- |
| Order 1  K= 13,83 | C | T(AT8-) | T(AT8+) |
| C |  | ns | *** |
| T(AT8-) |  |  | ns |
| T(AT8+) |  |  |  |
| Order 3  K= 8,595 | C | T(AT8-) | T(AT8+) |
| C |  | ns | * |
| T(AT8-) |  |  | ns |
| T(AT8+) |  |  |  |

| **Supplementary table 26\|Basal arbor branching segments surface area** | | | |
| --- | --- | --- | --- |
| Order 1  K= 23,68 | C | T(AT8-) | T(AT8+) |
| C |  | ns | *** |
| T(AT8-) |  |  | *** |
| T(AT8+) |  |  |  |
| Order 2  K= 7,506 | C | T(AT8-) | T(AT8+) |
| C |  | ns | ns |
| T(AT8-) |  |  | * |
| T(AT8+) |  |  |  |
| Order 3  K= 9,829 | C | T(AT8-) | T(AT8+) |
| C |  | ns | ns |
| T(AT8-) |  |  | * |
| T(AT8+) |  |  |  |

| **Supplementary table 27\|Basal arbor branching segments volume** | | | |
| --- | --- | --- | --- |
| Order 1  K= 31,24 | C | T(AT8-) | T(AT8+) |
| C |  | ns | *** |
| T(AT8-) |  |  | *** |
| T(AT8+) |  |  |  |
| Order 2  K= 13,21 | C | T(AT8-) | T(AT8+) |
| C |  | ns | * |
| T(AT8-) |  |  | ** |
| T(AT8+) |  |  |  |
| Order 3  K= 10,30 | C | T(AT8-) | T(AT8+) |
| C |  | ns | ns |
| T(AT8-) |  |  | * |
| T(AT8+) |  |  |  |

| **Supplementary table 28\|Basal arbor terminal segments tortuosity** | | | |
| --- | --- | --- | --- |
| Order 2  K= 8,423 | C | T(AT8-) | T(AT8+) |
| C |  | * | ns |
| T(AT8-) |  |  | ns |
| T(AT8+) |  |  |  |

| **Supplementary table 29\|Basal arbor terminal segments diameter** | | | |
| --- | --- | --- | --- |
| Order 2  K= 7,718 | C | T(AT8-) | T(AT8+) |
| C |  | ns | ns |
| T(AT8-) |  |  | * |
| T(AT8+) |  |  |  |
| Order 3  K= 6,737 | C | T(AT8-) | T(AT8+) |
| C |  | ns | ns |
| T(AT8-) |  |  | * |
| T(AT8+) |  |  |  |

| **Supplementary table 30\|Basal arbor terminal segments length** | | | |
| --- | --- | --- | --- |
| Order 2  K= 13,26 | C | T(AT8-) | T(AT8+) |
| C |  | ns | *** |
| T(AT8-) |  |  | ** |
| T(AT8+) |  |  |  |
| Order 3 K= 15,38 | C | T(AT8-) | T(AT8+) |
| C |  | ns | *** |
| T(AT8-) |  |  | *** |
| T(AT8+) |  |  |  |
| Order 4  K= 8,206 | C | T(AT8-) | T(AT8+) |
| C |  | ns | ns |
| T(AT8-) |  |  | * |
| T(AT8+) |  |  |  |

| **Supplementary table 31\|Basal arbor terminal segments surface area** | | | |
| --- | --- | --- | --- |
| Order 2  K= 20,03 | C | T(AT8-) | T(AT8+) |
| C |  | ns | *** |
| T(AT8-) |  |  | ** |
| T(AT8+) |  |  |  |
| Order 3 K= 18,35 | C | T(AT8-) | T(AT8+) |
| C |  | ns | *** |
| T(AT8-) |  |  | *** |
| T(AT8+) |  |  |  |
| Order 4  K= 7,582 | C | T(AT8-) | T(AT8+) |
| C |  | ns | ns |
| T(AT8-) |  |  | * |
| T(AT8+) |  |  |  |

| **Supplementary table 32\|Basal arbor terminal segments volume** | | | |
| --- | --- | --- | --- |
| Order 2  K= 20,95 | C | T(AT8-) | T(AT8+) |
| C |  | ns | *** |
| T(AT8-) |  |  | *** |
| T(AT8+) |  |  |  |
| Order 3  K= 16,76 | C | T(AT8-) | T(AT8+) |
| C |  | ns | *** |
| T(AT8-) |  |  | *** |
| T(AT8+) |  |  |  |

**Test used: Two way ANOVA and Bonferroni´s Multiple Comparison test.**

| **Supplementary table 33\| Main apical dendrite dendritic spine density** | | | |  |  |  |  |  |
| --- | --- | --- | --- | --- | --- | --- | --- | --- |
| Distance from soma (µm) | C vs T(AT8-) | C vs T(AT8+) | T(AT8-) vs T(AT8+) |  |  |  |  |  |
| 10 |  |  |  |  |  |  |  |  |
| 20 |  |  |  |  |  |  |  |  |
| 30 | ns | ns | ns |  |  |  |  |  |
| 40 | ns | ns | ns |  |  |  |  |  |
| 50 | ns | ns | ns |  |  |  |  |  |
| 60 | ns | ns | ns |  |  |  |  |  |
| 70 | ns | ns | ns |  |  |  |  |  |
| 80 | ns | ns | ns |  |  |  |  |  |
| 90 | ns | ns | ns |  |  |  |  |  |
| 100 | * | ns | ns |  |  |  |  |  |
| 110 | ns | ns | ns |  |  |  |  |  |
| 120 | ns | ns | ns |  |  |  |  |  |
| 130 | ns | ns | ns |  |  |  |  |  |
| 140 | ns | ns | ns |  |  |  |  |  |
| 150 | ns | ns | ns |  |  |  |  |  |
| 160 | ns | ns | ns |  |  |  |  |  |
| 170 | ns | ns | ns |  |  |  |  |  |
| 180 | ns | ns | ns |  |  |  |  |  |
|  |  |  |  |  |  |  |  |  |
| **Supplementary table 34\| Main apical dendrite dendritic spine length** | | | |  | **Supplementary table 35\| Main apical dendrite dendritic spine volume** | | | |
| Distance from soma (µm) | C vs T(AT8-) | C vs T(AT8+) | T(AT8-) vs T(AT8+) |  | Distance from soma (µm) | C vs T(AT8-) | C vs T(AT8+) | T(AT8-) vs T(AT8+) |
| 10 |  |  |  |  | 10 |  |  |  |
| 20 |  |  |  |  | 20 |  |  |  |
| 30 | ** | ns | ns |  | 30 | ns | ns | ns |
| 40 | ** | ns | ns |  | 40 | ns | ns | ns |
| 50 | ns | ns | ns |  | 50 | ns | ns | ns |
| 60 | *** | * | ns |  | 60 | ns | ns | ns |
| 70 | ** | ns | ns |  | 70 | ns | ns | ns |
| 80 | * | ns | ns |  | 80 | ns | ns | ns |
| 90 | ns | ns | ns |  | 90 | ns | ns | ns |
| 100 | ns | ns | ns |  | 100 | ns | ns | ns |
| 110 | ns | ns | ns |  | 110 | ns | ns | ns |
| 120 | ns | ns | ns |  | 120 | ns | ns | ns |
| 130 | ** | ns | ns |  | 130 | ns | ns | ns |
| 140 | ns | ns | ns |  | 140 | ns | ns | ns |
| 150 | ns | ns | ns |  | 150 | ns | * | ns |
| 160 | ns | ns | ns |  | 160 | ns | ns | ns |
| 170 | ns | ns | ns |  | 170 | ns | ns | ns |
| 180 | ns | ns | ns |  | 180 | ns | ns | ns |
| **Supplementary table 36\| Basal dendrites dendritic spine length** | | | |  | **Supplementary table 38\| Basal dendrites dendritic spine volume** | | | |
| Distance from soma (µm) | C vs T(AT8-) | C vs T(AT8+) | T(AT8-) vs T(AT8+) |  | Distance from soma (µm) | C vs T(AT8-) | C vs T(AT8+) | T(AT8-) vs T(AT8+) |
| 10 |  |  |  |  | 10 |  |  |  |
| 20 | ns | ns | ** |  | 20 | * | ns | ns |
| 30 | *** | ns | ** |  | 30 | ** | ns | ns |
| 40 | ns | ns | ns |  | 40 | *** | ns | * |
| 50 | * | ns | ns |  | 50 | ns | ns | ns |
| 60 | ns | ns | ns |  | 60 | ** | ns | ns |
| 70 | ns | ns | ns |  | 70 | ns | ns | ns |
| 80 | ns | ns | ns |  | 80 | * | ns | ns |
| 90 | ns | ns | ns |  | 90 | * | ns | ns |
| 100 | ns | ns | ns |  | 100 | ns | ns | ns |
| 110 | ns | ns | ns |  | 110 | ns | ns | ns |
| 120 | ns | ns | ns |  | 120 | * | ns | ns |
| 130 | ns | ns | ns |  | 130 | ** | ns | ns |
| 140 | ns | ns | ns |  | 140 | ns | ns | ns |
| 150 | ns | ns | ns |  | 150 | ns | ns | ns |

| **Supplementary table 37\| Basal dendrites dendritic spine area** | | | |  |
| --- | --- | --- | --- | --- |
| Distance from soma (µm) | C vs T(AT8-) | C vs T(AT8+) | T(AT8-) vs T(AT8+) |  |
| 10 |  |  |  |  |
| 20 | ** | ns | ns |  |
| 30 | *** | ns | * |  |
| 40 | *** | ns | * |  |
| 50 | ** | ns | ns |  |
| 60 | ** | ns | ns |  |
| 70 | ns | ns | ns |  |
| 80 | * | ns | ns |  |
| 90 | ** | ns | ns |  |
| 100 | ns | ns | ns |  |
| 110 | ns | ns | ns |  |
| 120 | * | ns | ns |  |
| 130 | ** | ns | ns |  |
| 140 | ns | ns | ns |  |
| 150 | ns | ns | ns |  |

**Mean values calculated per dendrite. Tests used: Kruskal-Wallis and Dunn´s Multiple Comparison test for non-parametric data and One way ANOVA and Bonferroni´s Multiple Comparison test for parametric data.**

| **Supplementary table 39\| Main apical dendrite dendritic spine length** | | | |
| --- | --- | --- | --- |
|  | C | T(AT8-) | T(AT8+) |
| C |  | *** | ** |
| T(AT8-) |  |  | ns |
| T(AT8+) |  |  |  |
| **Supplementary table 40\| Main apical dendrite dendritic spine area** | | | |
|  | C | T(AT8-) | T(AT8+) |
| C |  | ** | ** |
| T(AT8-) |  |  | ns |
| T(AT8+) |  |  |  |
| **Supplementary table 41\| Main apical dendrite dendritic spine volume** | | | |
|  | C | T(AT8-) | T(AT8+) |
| C |  | ns | ** |
| T(AT8-) |  |  | ns |
| T(AT8+) |  |  |  |

| **Supplementary table 42\| Basal dendrites dendritic spine length** | | | |
| --- | --- | --- | --- |
|  | C | T(AT8-) | T(AT8+) |
| C |  | *** | ns |
| T(AT8-) |  |  | ns |
| T(AT8+) |  |  |  |
| **Supplementary table 43\| Basal dendrites dendritic spine area** | | | |
|  | C | T(AT8-) | T(AT8+) |
| C |  | *** | * |
| T(AT8-) |  |  | ** |
| T(AT8+) |  |  |  |
| **Supplementary table 44\| Basal dendrites dendritic spine volume** | | | |
|  | C | T(AT8-) | T(AT8+) |
| C |  | *** | * |
| T(AT8-) |  |  | ns |
| T(AT8+) |  |  |  |

Supplementary table legends:

Suppl. Table 1: Sholl analysis statistical significance of the number of intersections in the apical arbor.

Suppl. Table 2: Mean total values statistical significance of the number of intersections in the apical arbor.

Suppl. Table 3: Mean total values statistical significance of the number of nodes in the apical arbor.

Suppl. Table 4: Sholl analysis statistical significance of the number ofendings in the apical arbor.

Suppl. Table 5: Mean total values statistical significance of the number of endings in the apical arbor.

Suppl. Table 6: Sholl analysis statistical significance of the average diameter in the apical arbor.

Suppl. Table 7: Sholl analysis statistical significance of dendritic length in the apical arbor.

Suppl. Table 8: Sholl analysis statistical significance of dendritic surface area in the apical arbor.

Suppl. Table 9: Sholl analysis statistical significance of the number of nodes in the basal arbor.

Suppl. Table 10: Sholl analysis statistical significance of the average diameter in the basal arbor.

Suppl. Table 11: Mean total values statistical significance of the average diameter in the basal arbor.

Suppl. Table 12: Sholl analysis statistical significance of dendritic surface area in the basal arbor.

Suppl. Table 13: Sholl analysis statistical significance of the dendritic volume in the basal arbor.

Suppl. Table 14: Mean total values statistical significance of the dendritic volume in the basal arbor.

Suppl. Table 15: Statistical significance of segment analysis for branching segments tortuosity in the apical arbor.

Suppl. Table 16: Statistical significance of segment analysis for branching segments dendritic length in the apical arbor.

Suppl. Table 17: Statistical significance of segment analysis for branching segments dendritic surface area in the apical arbor.

Suppl. Table 18: Statistical significance of segment analysis for branching segments dendritic volume in the apical arbor.

Suppl. Table 19: Statistical significance of segment analysis for terminal segments tortuosity in the apical arbor.

Suppl. Table 20: Statistical significance of segment analysis for terminal segments dendritic length in the apical arbor.

Suppl. Table 21: Statistical significance of segment analysis for terminal segments dendritic surface area in the apical arbor.

Suppl. Table 22: Statistical significance of segment analysis for terminal segments dendritic volume in the apical arbor.

Suppl. Table 23: Statistical significance of segment analysis for branching segments tortuosity in the basal arbor.

Suppl. Table 24: Statistical significance of segment analysis for branching segments dendritic diameter in the basal arbor.

Suppl. Table 25: Statistical significance of segment analysis for branching segments dendritic length in the basal arbor.

Suppl. Table 26: Statistical significance of segment analysis for branching segments dendritic surface area in the basal arbor.

Suppl. Table 27: Statistical significance of segment analysis for branching segments dendritic volume in the basal arbor.

Suppl. Table 28: Statistical significance of segment analysis for terminal segments tortuosity in the basal arbor.

Suppl. Table 29: Statistical significance of segment analysis for terminal segments dendritic diameter in the basal arbor.

Suppl. Table 30: Statistical significance of segment analysis for terminal segments dendritic length in the basal arbor.

Suppl. Table 31: Statistical significance of segment analysis for terminal segments dendritic surface area in the basal arbor.

Suppl. Table 32: Statistical significance of segment analysis for terminal segments dendritic volume in the basal arbor.

Suppl. Table 33: Sholl analysis statistical significance for dendritic spine density of the apical main dendrite.

Suppl. Table 34: Sholl analysis statistical significance for dendritic spine length of the apical main dendrite.

Suppl. Table 35: Sholl analysis statistical significance for dendritic spine volume of the apical main dendrite.

Suppl. Table 36: Sholl analysis statistical significance for dendritic spine length of the basal dendrites.

Suppl. Table 37: Sholl analysis statistical significance for dendritic spine area of the basal dendrites.

Suppl. Table 38: Sholl analysis statistical significance for dendritic spine volume of the basal dendrites.

Suppl. Table 39: Mean total values statistical significance of the average dendritic spine length in the apical main dendrite.

Suppl. Table 40: Mean total values statistical significance of the average dendritic spine area in the apical main dendrite.

Suppl. Table 41: Mean total values statistical significance of the average dendritic spine volume in the apical main dendrite.

Suppl. Table 42: Mean total values statistical significance of the average dendritic spine length in the basal dendrites.

Suppl. Table 43: Mean total values statistical significance of the average dendritic spine area in the basal dendrites.

Suppl. Table 44: Mean total values statistical significance of the average dendritic spine volume in the basal dendrites.


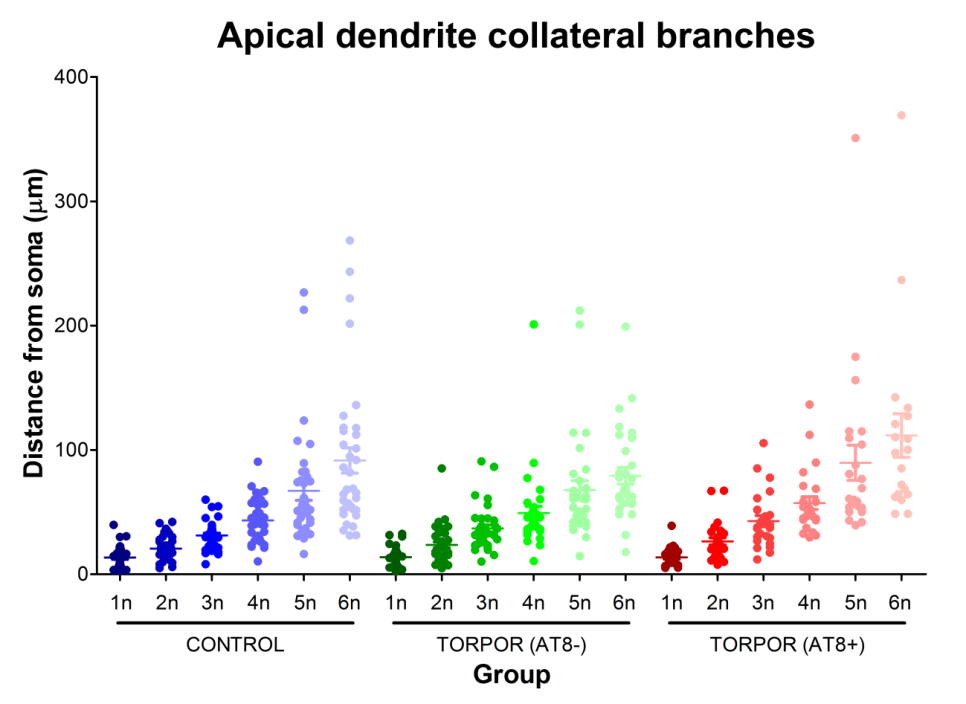


Supplementary Figure 1. Graph showing the distance from soma at which the first 6 nodes of the main apical dendrite are located for control (blue), T(AT8-) (green) and T(AT8+) (red) groups.
